# Comparative Genome Analysis Provides Insights into the Evolution and Adaptation of *Pseudomonas syringae* pv. *aesculi* on *Aesculus hippocastanum*

**DOI:** 10.1371/journal.pone.0010224

**Published:** 2010-04-19

**Authors:** Sarah Green, David J. Studholme, Bridget E. Laue, Federico Dorati, Helen Lovell, Dawn Arnold, Joan E. Cottrell, Stephen Bridgett, Mark Blaxter, Edgar Huitema, Richard Thwaites, Paul M. Sharp, Robert W. Jackson, Sophien Kamoun

**Affiliations:** 1 Centre for Forestry and Climate Change, Forest Research, Roslin, Midlothian, United Kingdom; 2 The Sainsbury Laboratory, Norwich, Norfolk, United Kingdom; 3 School of Biological Sciences, The University of Reading, Reading, Berkshire, United Kingdom; 4 Centre for Research in Plant Science, University of the West of England, Bristol, Avon and Somerset, United Kingdom; 5 Centre for Human and Ecological Sciences, Forest Research, Roslin, Midlothian, United Kingdom; 6 The GenePool Genomics Facility, University of Edinburgh, Edinburgh, Lothian and Borders, United Kingdom; 7 Institute of Evolutionary Biology, University of Edinburgh, Edinburgh, Lothian and Borders, United Kingdom; 8 The Food and Environment Research Agency, Sand Hutton, Yorkshire, United Kingdom; University of Poitiers, France

## Abstract

A recently emerging bleeding canker disease, caused by *Pseudomonas syringae* pathovar *aesculi* (*Pae*), is threatening European horse chestnut in northwest Europe. Very little is known about the origin and biology of this new disease. We used the nucleotide sequences of seven commonly used marker genes to investigate the phylogeny of three strains isolated recently from bleeding stem cankers on European horse chestnut in Britain (E-*Pae*). On the basis of these sequences alone, the E-*Pae* strains were identical to the *Pae* type-strain (I-*Pae*), isolated from leaf spots on Indian horse chestnut in India in 1969. The phylogenetic analyses also showed that *Pae* belongs to a distinct clade of *P. syringae* pathovars adapted to woody hosts. We generated genome-wide Illumina sequence data from the three E-*Pae* strains and one strain of I*-Pae*. Comparative genomic analyses revealed pathovar-specific genomic regions in *Pae* potentially implicated in virulence on a tree host, including genes for the catabolism of plant-derived aromatic compounds and enterobactin synthesis. Several gene clusters displayed intra-pathovar variation, including those encoding type IV secretion, a novel fatty acid biosynthesis pathway and a sucrose uptake pathway. Rates of single nucleotide polymorphisms in the four *Pae* genomes indicate that the three E-*Pae* strains diverged from each other much more recently than they diverged from I-*Pae*. The very low genetic diversity among the three geographically distinct E-*Pae* strains suggests that they originate from a single, recent introduction into Britain, thus highlighting the serious environmental risks posed by the spread of an exotic plant pathogenic bacterium to a new geographic location. The genomic regions in *Pae* that are absent from other *P. syringae* pathovars that infect herbaceous hosts may represent candidate genetic adaptations to infection of the woody parts of the tree.

## Introduction

In recent decades there has been an unprecedented rise in cases of exotic or previously unknown invasive plant diseases emerging in new ecosystems, posing a threat to food security and to urban as well as rural plant communities [1]. The rise in mobility of human populations and increased global commerce, for example in the international plant trade, have likely contributed to the spread of these pathogens to new geographical areas where previously unexposed plants have not yet evolved specific resistance [2]. Usually, little information is available on the origin, biology and genetics of these newly arising diseases during the early stages of an epidemic. Comparative genomics, based on an ever-increasing number of complete genome sequences, can be used to reveal numerous insights into host-pathogen interactions, the evolution of pathogenic lifestyles and adaptation to new niches [3]. Due to the recent developments in genomics technology, it is becoming almost routine to sequence emerging prokaryotic pathogens of humans [4]. However, genomics tools have not been rapidly and systematically applied to emerging plant pathogens, therefore hindering the opportunity to gain useful insights into the biology of emerging plant diseases.

**Figure 1:**
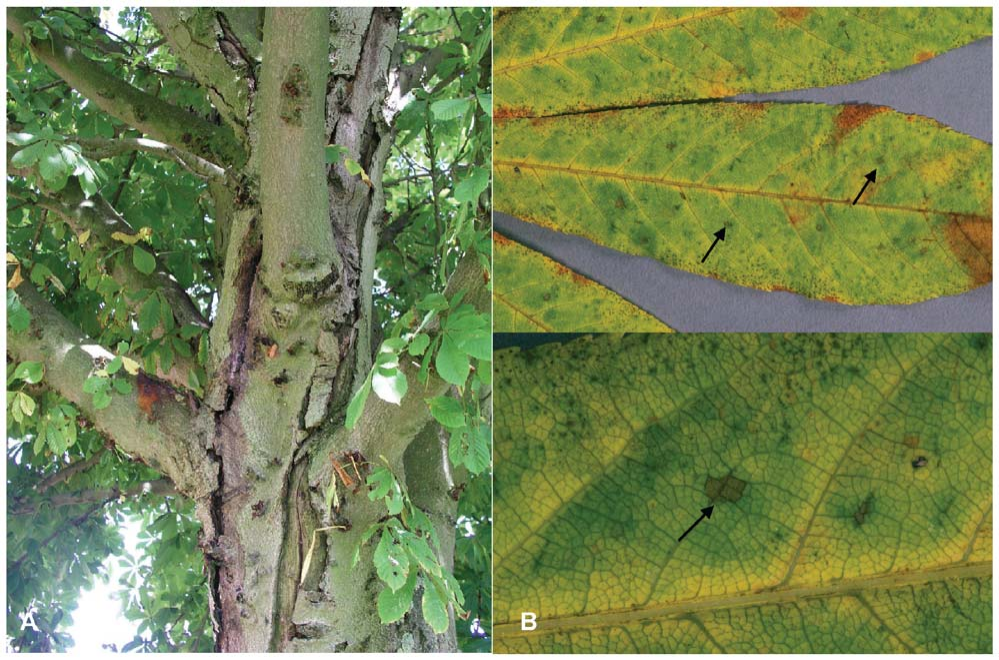
Disease symptoms of *Pae* on horse chestnut. (A) Bleeding canker on stem of European horse chestnut caused by E-*Pae* and (B) leaf spots (arrows) on Indian horse chestnut caused by I-*Pae*.

Bleeding canker of European horse chestnut (*Aesculus hippocastanum*) is a destructive new disease which was first noticed in 2002/2003. The disease is currently affecting hundreds of thousands of European horse chestnut trees across several countries in northwest Europe, resulting in severe damage to rural and urban amenity landscapes [5], [6]. Disease symptoms include bleeding cankers located on the stem and branches, foliar discoloration, and crown dieback often leading to tree death [6]. In 2007, over 70% of horse chestnut trees surveyed in parts of England exhibited symptoms typical of bleeding canker disease, with 36% and 42% of surveyed trees showing these symptoms in Wales and Scotland, respectively [7]. The causal agent responsible for this new epidemic has only recently been identified as the Gram-negative fluorescent bacterium, *Pseudomonas syringae* pathovar *aesculi* (*Pae*). This identification was based on a partial sequence for its gyrase B gene, which was identical to that of the *Pae* type strain isolated from leaf spot lesions on Indian horse chestnut (*Aesculus indica*) from the Himachal Pradesh, Northern India in 1969. Prior to the European epidemic, this was the only location where *Pae* had been reported [5], [6], [8]–[10]. This suggests that *Pae* may have originated from India and been recently introduced into Europe. If this is indeed the case, *Pae* has found a new host, European horse chestnut, on which it is highly mobile and aggressive, causing frequently lethal stem cankers (Figure 1A) that contrast with the minor leaf lesions observed on Indian horse chestnut (Figure 1B). This emerging disease has become an important tree health issue in Great Britain, attracting intense and broad public attention due to its dramatic impact on a tree species of such high amenity and cultural value.

The *Pae* epidemic has highlighted gaps in our general understanding of the biology of bacterial diseases of trees. There are at least 50 pathovars of the species *Pseudomonas syringae*, which can be distinguished by host range, and which infect a wide range of mostly herbaceous but also some woody plants. Almost nothing is known about the biology of *Pae* on European horse chestnut, including the reasons for its apparently sudden emergence, the genetic factors contributing to its observed high levels of virulence on a woody host or its evolutionary relationships to other strains of *P. syringae*. Due to its aggressiveness and rapidity of spread throughout a high proportion of the European horse chestnut population in affected countries, *Pae* presents an excellent model system for gaining a greater understanding of bacterial tree diseases. Due to the economic importance of *P. syringae* pathovars and their value as models for studying plant pathogenesis, complete genome sequence data are available for three pathovars on herbaceous hosts, tomato and bean; *P. syringae* pv. *tomato* DC300 (*Pto* DC3000), *P. syringae* pv. *syringae* (*Psy* B728a) and *P. syringae* pv. *phaseolicola* (*Pph* 1448A) [11]–[13]. Draft genome sequences are also available for two other pathovars; *P. syringae* pv. *oryzae* (*Por*1-6) pathogenic on rice and *P. syringae* pv. *tabaci* (*Pta* 11528) which causes disease on wild tobacco [14], [15]. These complete and draft *P. syringae* genome sequences provide important reference sequences for a comparative genomic study of *Pae*.

We have generated good quality draft genome sequences for a strain of *Pae* recently isolated from a bleeding canker on diseased European horse chestnut in Britain (strain 2250) as well as the Indian type strain of *Pae* that causes a leaf-spot disease on Indian horse chestnut [8], [9]. We also generated whole-genome re-sequencing data for two additional *Pae* strains (P6617 and P6623) from different geographical locations in Britain. These are the first reported whole-genome sequences for pseudomonad pathogens of a woody host. The aim of this study was to gain insights into the biology and evolution of *Pae* strains causing the current disease epidemic on European horse chestnut. We achieved this by comparing the *Pae* genome with sequences from other *P. syringae* pathovars and by determining the genomic variation among all four *Pae* strains. We show that *Pae* belongs to a distinct clade of *P. syringae* pathovars that specialise in infecting woody hosts. *Pae* harbors genomic regions that are absent from other *P. syringae* pathovars that infect herbaceous hosts and represent candidate genetic adaptations to pathogenicity on woody parts of the tree. Comparison of sequences in the core genome reveals that the British *Pae* strains are very closely related and, most likely, descend from a single, recent introduction into Britain. Their relationship with Indian *Pae* is more distant but sufficiently close that they share nucleotide sequence identity over seven house-keeping genes. However, despite the close relationship between the British and Indian strains of *Pae*, their genomes display marked differences resulting from loss and/or gain of a range of genes since their divergence.

## Results

### Genome-wide sequence data

We generated genome-wide Illumina [16] sequence data from three strains of *Pae* recently isolated from diseased European horse chestnut trees in Britain (henceforth referred to as E-*Pae*). These included E-*Pae* 2250 (from Pitlochry, Perthshire, Scotland, 2008), E-*Pae* P6617 (from Glasgow, Strathclyde, Scotland, 2006) and E-*Pae* P6623 (from Farnham, Surrey, England, 2006). We also generated genome-wide Illumina sequence data from the type-strain, *Pae* NCPPB3681, originally isolated from Indian horse chestnut in India in 1969 (henceforth referred to as I-*Pae*). Of the three E-*Pae* strains, 2250 generated the highest quality sequence and this, along with that of I-*Pae*, was used to generate draft *de novo* genome assemblies as described in MATERIALS AND METHODS. Thus, unless otherwise indicated, E-*Pae* refers to the 2250 strain assembly.

The E-*Pae* genome assembly (strain 2250) yielded 776 contigs comprising 364 scaffolds (maximum scaffold length  = 190 kb; N_50_ scaffold length  = 42.5 kb). The sum of the contig lengths for E-*Pae* was 5,926,327 nucleotides, which is approximately the expected size based on previously sequenced *P. syringae* genomes, and the assembly contained 5,621 predicted protein-coding genes. The I-*Pae* genome assembly yielded 841 contigs comprising 557 scaffolds (maximum scaffold length  = 93 kb; N_50_ scaffold length  = 26.4 kb). The sum of the contig lengths for I-*Pae* was 5,895,455 nucleotides and the assembly contained 5,683 predicted protein-coding genes. The sequences of the assemblies have been deposited in GenBank with accession numbers ACXT00000000 (E-*Pae*) and ACXS00000000 (I-*Pae*).

### *Pae* belongs to a distinct clade of pathogens of woody hosts

**Figure 2:**
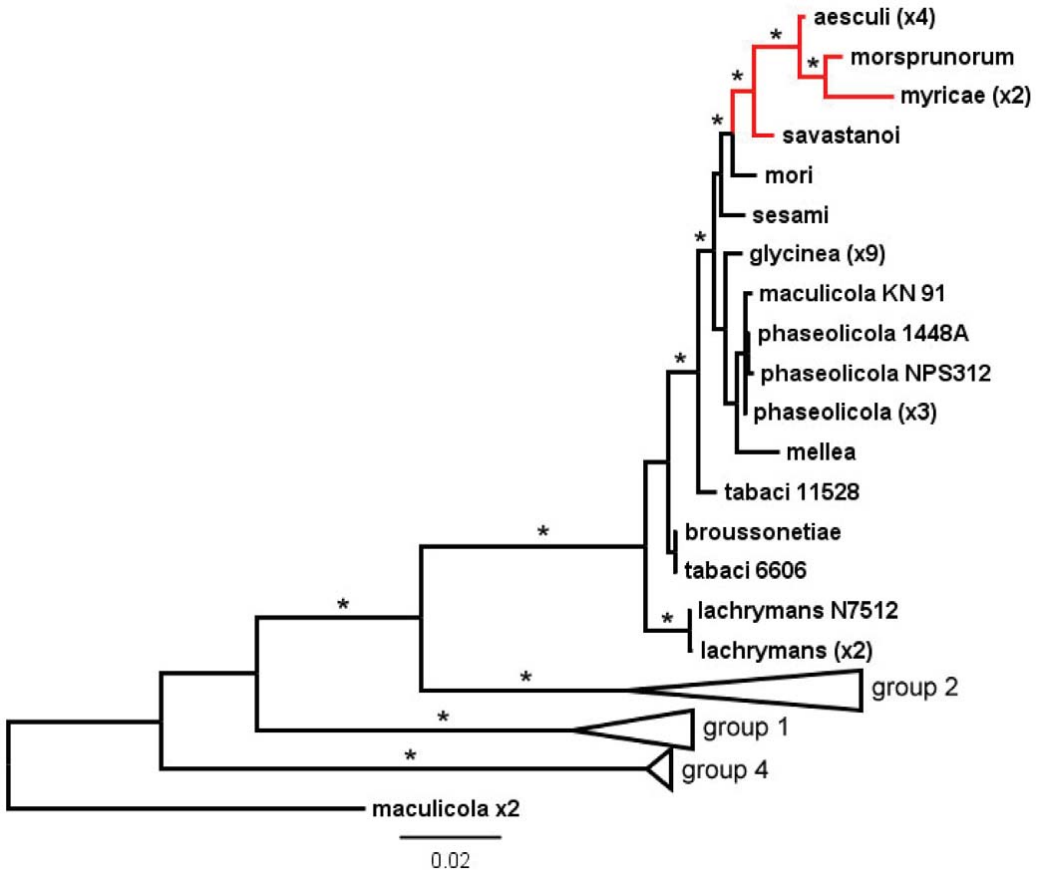
Evolutionary relationship of *P. syringae* pv. *aesculi* to other strains of *P. syringae*. Phylogenetic relationships were estimated from concatenated sequences from seven housekeeping genes (3129 bp) using a Bayesian Markov chain Monte Carlo method (See MATERIALS AND METHODS). Values in brackets indicate numbers of strains of the same pathovar with identical sequences (*e.g.*, four strains of *Pae*). Red branches indicate the clade comprised of four pathovars that infect a woody host. Stars mark internal branches supported by posterior probability values of at least 0.98. The scale bar represents 0.02 nucleotide substitutions per site. Details are shown only for the clade designated as group 3 by [17], which corresponds to genomospecies 2 [18]; group 2 contains genomospecies 1 strains including *P. syringae* pv. *syringae*, group 1 contains genomospecies 3 strains including *P. syringae* pv. *tomato*, and group 4 contains probable genomospecies 4 strains including *P. syringae* pv. *oryzae*.

The evolutionary relationships among numerous *P. syringae* pathovars (but not including *Pae*) have previously been investigated using the concatenated sequences of seven housekeeping genes [17]. We extracted the orthologous sequences from the four *Pae* genome assemblies and found that the four strains were identical over the alignment of 3,129 nucleotides. Our phylogenetic analyses (Figure 2) placed *Pae* within a major lineage referred to as group 3 in [17], and apparently corresponding to genomospecies 2 as defined by earlier DNA-DNA hybrization studies [18]. Interestingly, most of the strains within group 3 and genomospecies 2 are associated with herbaceous hosts. However, *Pae* fell within a statistically well-supported clade (Figure 2) comprised of pathovars *morsprunorum*, *myricae*, *savastanoi* and *mori*; these are pathogens of apricot, bayberry, olive and mulberry, respectively, which are all woody plants.

### Evolutionary relationships among *Pae* strains

On the basis of the seven previously used housekeeping gene sequences, the three E-*Pae* strains and the single I-*Pae* strain were identical. However, the genome-wide Illumina sequence data allowed us to undertake a comprehensive search for genetic variation among the strains over three megabases of genome for which there was sufficiently deep coverage and unambiguous Illumina data from all four strains

The sequences of the three E-*Pae* strains isolated from the woody parts of European horse chestnut differed at only three nucleotides over the three megabases. Recent analyses of the rates of short-term evolution in *Neisseria* [19], *Helicobacter* [20] and *Campylobacter* [21] have all yielded estimated mutation rates in the range 3-5×10^−5^ substitutions per site per year. The E-*Pae* strains differed at 0-6×10^−7^ substitutions per site. Therefore, unless *P. syringae* accumulates nucleotide substitutions at a rate several orders of magnitude slower than these other bacteria, our data indicate that the E-*Pae* strains share a very recent common ancestor and are descended from a single introduction into Britain.

I-*Pae* differed from E-*Pae* at 1,613 nucleotides over the same three megabases of the genome. While this reflects a very low level of divergence (only 5×10^−4^ nucleotide substitutions per site) it nevertheless indicates that the common ancestry of E-*Pae* and I-*Pae* occurred long before the divergence of the E-*Pae* strains from each other.

### *Pae* harbors pathovar-specific genomic regions of potential importance in adaptation to *Aesculus*

The draft genomes of E-*Pae* and I-*Pae* showed greatest sequence similarity to *Pph* 1448A and *Pta* 11528 when compared with the genomes of other previously sequenced *P. syringae* strains from herbaceous hosts, based on genome-wide MUMMER alignments. This is consistent with the results of the phylogenetic analysis (Figure 2) based on the seven housekeeping genes. Based on MUMMER alignments, approximately 15% of the E-*Pae* and 15% of the I-*Pae* genomes were not conserved in *Pph* 1448A or *Pta* 11528. Therefore, we hypothesised that the *Pae* genomes might contain sequences specifically related to their association with a tree host. We identified 85 genomic regions from E-*Pae* (each between 1 kb and 23 kb long) that showed no detectable nucleotide sequence similarity to *Pph* 1448A, *Pta* 11528, *Pto* DC3000, *Psy* B728a, nor *Por* 1_6. The total length of these regions was 270 kb. We also identified 307 kb of sequences in I-*Pae* that had no similarity to the sequenced genomes of other pathovars. Some of these genomic regions currently found only in *Pae* could be implicated in fitness on a tree host. These are examined in greater detail in the following sections.

### Catabolism of phenolic compounds

**Figure 3:**
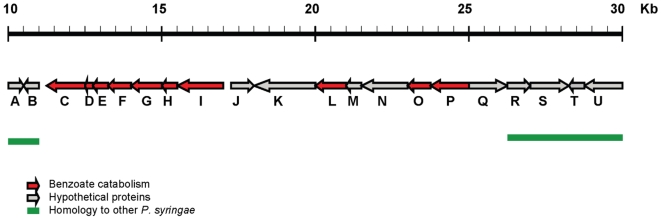
An E-*Pae* encoded pathway for the catabolism of plant-derived aromatic compounds. Shown is a 20 kb section of a 46 kb E-*Pae* contig (GenBank: ACXT01000012) which contains putative genes encoding enzymes for the catabolism of benzoate *via* the catechol branch of the *β*-ketoadipate pathway (Indicated by red arrows). Full details of the predicted genes based on blastp searches are shown in Table 1. Regions of sequence identity with other *P. syringae* genomes (with a significance threshold of 1e-10) are indicated by the green bars. Grey arrows indicate uncharacterized proteins.

Of considerable significance was the presence of a 46 kb contig (GenBank: ACXT01000012) in E-*Pae*, most of which was conserved in I-*Pae*, that shared no nucleotide sequence similarity with sequenced genomes of other *P. syringae* pathovars over most of its length and contained predicted genes for the catabolism of phenolic compounds (Figure 3, Table 1). Eight predicted proteins coded for by this region in E-*Pae* (0368 to 0374 and 0381) had greatest amino acid sequence identities with enzymes involved in the catabolism of benzoate *via* the catechol branch of the *β*-ketoadipate pathway [22] found in soil-inhabiting, decomposing bacteria including *Acinetobacter* spp. and *Pseudomonas putida* (Table 1). In addition, proteins encoded by genes 0377 and 0380 on this contig were also likely to be involved in the catabolism of phenolic compounds (Table 1).

**Figure 4:**
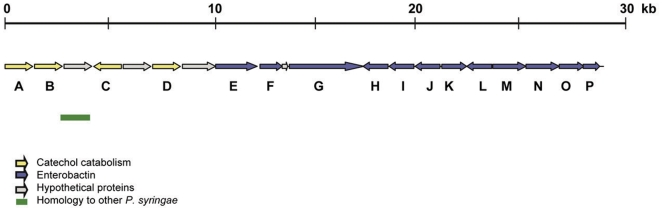
E-*Pae-*encoded pathways for the catabolism of plant-derived aromatic compounds and enterobactin biosynthesis. Shown is a 27 kb E-*Pae* contig (Genbank: ACXT01000075) which includes genes encoding the protocatechuate 4,5-dioxygenase pathway (yellow arrows) as well as a pathway for enterobactin biosynthesis (blue arrows). Full details of the predicted genes based on blastp searches are shown in Table 2. Regions of sequence identity with other *P. syringae* genomes (with a significance threshold of 1e-10) are indicated by the green bars. Uncharacterized or hypothetical proteins are indicated by grey arrows.

Another contig in E*-Pae* encoded four predicted proteins (genes 1439, 1440, 1442, 1444; located on a 27 kb contig, GenBank: ACXT01000075) that were present in I-*Pae*, but not conserved in other sequenced *P. syringae* pathovars associated with herbaceous hosts (Figure 4, Table 2). These four proteins showed homology to enzymes involved in protocatechuate degradation via the protocatechuate 4,5-dioxygenase pathway and included amongst them the iron-requiring *β*-subunit of protocatechuate 4,5-dioxygenase (Table 2).

**Table 1:** Predicted proteins in E-*Pae* that may be involved in the catabolism of plant-derived aromatic compounds *via* the catechol branch of the *β*-ketoadipate pathway.

ORF	Protein locus tag	Predicted function	Amino acid identity	Species of best BLASTP match in SwissProt
C	PSAESCULI2250_0368	Catechol 1,2-dioxygenase	58%	*Acinetobacter* sp.
D	PSAESCULI2250_0369	Muconolactone delta-isomerase	75%	*P. putida*
E	PSAESCULI2250_0370	Muconate cycloisomerase C-terminal	72%	*P. putida*
F	PSAESCULI2250_0371	Muconate cycloisomerase N-terminal	72%	*P. putida*
G	PSAESCULI2250_0372	Benzoate 1,2-dioxygenase electron transfer component	65%	*P. fluorescens*
H	PSAESCULI2250_0373	Benzoate 1,2-dioxygenase beta subunit	39%	*Acinetobacter* sp.
I	PSAESCULI2250_0374	Benzoate 1,2 -dioxygenase alpha subunit	44%	*Acinetobacter* sp.
L	PSAESCULI2250_0377	Protein involved with the meta pathway of phenol degradation	66%	*Acinetobacter* sp.
O	PSAESCULI2250_0380	Short chain dehydrogenase	68%	*Acinetobacter* sp.
P	PSAESCULI2250_0381	Dienelactone hydrolase	38%	*P. putida*

These proteins are encoded on a 30 Kb region of a 45.9 Kb contig (GenBank: ACXT01000012), as depicted in Figure 3.

### Iron acquisition

Both E-*Pae* and I-*Pae* encoded a number of genes involved in iron acquisition that have not yet been found in other pathovars of *P*. *syringae* associated with herbaceous hosts (Figure 4, Table 2). Most prominent was a gene cluster related to the enterobactin (Ent) siderophore biosynthesis genes *entABEC*, an Ent import component related to FepB, a homologue of the enterobactin exporter (EntS), and the esterase (Fes) involved in ferri-enterobactin dissociation (E-*Pae* genes 1447, 1449 and 1453-1458 on GenBank: ACXT01000075; I-*Pae* genes 2794-2799 on GenBank: ACXT01000181; 3668 and 3670 on GenBank: ACXT01000216). BLASTP analyses of the *entABEC*-encoded proteins in *Pae* found the highest protein identities in the soil dwelling bacteria *Pseudomonas entomophila* (EntE, 59%; EntC, 55%) and *Azotobacter vinelandii* (EntA, 50%; EntB, 48%; EntE, 50%; EntC, 47%) (Table 2). Interestingly, this novel enterobactin gene cluster lies on the same 27 kb contig (GenBank: ACXT01000075) and in close proximity to the three proteins involved in the protocatechuate 4,5-dioxygenase pathway (described above) in E-*Pae* and I-*Pae* (Figure 4).

**Table 2:** Predicted proteins in E-*Pae* that may be involved in the catabolism of plant-derived aromatic compounds *via* the protocatechuate 4,5-dioxygenase pathway (A–D) and enterobactin synthesis (F–P).

ORF	Locus tag	Predicted function	Amino acid identity	Species of best BLASTP match in Swiss-Prot
		**Protocatechuate 4,5-dioxygenase pathway**		
A	PSAESCULI2250_1439	4-hydroxybenzoate transporter protein	48%	*P. putida*
B	PSAESCULI2250_1440	beta-subunit of protocatechuate 4,5-dioxygenase	39%	*Sphingomonas paucimobilis*
C	PSAESCULI2250_1442	LysR transcriptional regulator	80%	*P. putida*
D	PSAESCULI2250_1444	4-carboxy-4-hydroxy-2-oxoadipate aldolase	82%	*Azotobacter vinelandii*
		**Enterobactin biosynthesis**		
E	PSAESCULI2250_1446	TonB siderophore receptor, iron uptake	69%	*P. putida*
F	PSAESCULI2250_1447	Ferric enterobactin esterase	51%	*Serratia maculans*
G	PSAESCULI2250_1449	Enterobactin synthase	63%	*Serratia maculans*
H	PSAESCULI2250_1450	Enterobactin transporter	76%	*Serratia maculans*
I	PSAESCULI2250_1451	Enterobactin synthase	63%	*E. coli*
J	PSAESCULI2250_1452	Enterobactin transport	64%	*E. coli*
K	PSAESCULI2250_1453	Enterobactin exporter	62%	*E. coli*
L	PSAESCULI2250_1454	Enterobactin binding protein	63%	*E. coli*
M	PSAESCULI2250_1455	Isochorismate synthase	46%	*E. coli*
N	PSAESCULI2250_1456	Enterobactin synthetase	60%	*E. coli*
O	PSAESCULI2250_1457	Enterobactin synthetase	57%	*E. coli*
P	PSAESCULI2250_1458	Siderophone biosynthesis	57%	*E. coli*

These proteins are encoded by a 27.2 kb contig (GenBank: ACXT01000075) as depicted in Figure 4.

### Nitric oxide metabolism

We identified two genes with a predicted function in nitric oxide metabolism, which are conserved in both E-*Pae* (genes 0518 and 0519 on GenBank:ACXT01000019); see Figure S1), and I-*Pae* (genes 3361 and 3362 on GenBank: ACXT01000267) but which are not present in other sequenced *P. syringae* pathovars. These genes may have a role in protection of *Pae* from host defence responses [23], [24]. The predicted product of E-*Pae* gene 0518 shared 61% amino acid sequence identity with a nitric oxide (NO) dioxygenase from *Pseudomonas aeruginosa* that converts NO to NO_3_^−^, and gene 0519 shared 48% identity with the σ^54^-dependent nitric oxide reductase transcription regulator NorR [25], from the denitrifying bacterium *Ralstonia eutropha*, which reduces NO to N_2_O under anaerobic conditions. A canonical σ^54^-binding site sequence is also located upstream of this gene, consistent with it being the regulatory target of the NorR homologue.

### Secondary metabolism

A 10 kb genomic region, conserved in both E-*Pae* (genes 0811-0819 on GenBank: ACXT01000515) and I-*Pae* (genes 1753-1761 on GenBank: ACXS01000161) but not in other sequenced *P. syringae* pathovars, appeared to encode a secondary metabolism pathway involved in the production of a toxin. E-*Pae* gene 0812 shared 21% amino acid sequence identity with a novel redox protein toxin (CADD), previously only known in *Chlamydia* spp., that contains a di-iron centre and has been implicated in the modulation of host cell apoptosis [26]. The protein product of E-*Pae* gene 0814 shared 26% identity with an alkyl hydroperoxide reductase (Swiss-Prot: P26829). Other genes in the cluster encoded protein sequences with similarity to aminotransferases (0813), dioxygenases (0815) and acetylornithine deacetylase (0818). The cluster also encoded a transcriptional regulator (0811) and a major facilitator superfamily (MFS) transporter (0819) that might be involved in regulation of the pathway and transport of a product or substrate.

### E-*Pae* and I-*Pae* show genomic differences implicated in host association and fitness

Given that E-*Pae* was isolated from cankers on woody organs and I-*Pae* from leaf spots, we expected to find strain-specific genes and gene clusters that may reflect their different mechanisms of pathogenesis. Comparisons between the sequence assemblies of E-*Pae* and I-*Pae* revealed several differences, which included Type III secretion system (T3SS) proteins and factors implicated in fitness (Table 3). There were a number of genomic regions present in I-*Pae* but absent from E-*Pae*, including two Type VI secretion systems (T6SS) (Tables S1. S2), a microcin gene cluster (Figure S2) and a novel methionine sulphoxide (Table 3), which are described in more detail in Text S1. Of greater interest were a number of genes and pathways which were present in E-*Pae* and absent from I-*Pae* which might reflect adaptation to the woody parts of the tree.

**Table 3:** Examples of intra-pathovar variation within *Pae* in terms of presence or absence of genes.

Predicted functions of gene products (Refseq protein locus tags/accession numbers)	I-*Pae*	E-*Pae* 2250	E-*Pae* P6617	E-*Pae* P6623
Conjugal transfer protein (PSPPH_B0041)	**-**	**+**	**-**	**+**
Killer protein (PSPPH_B0042);	**-**	**+**	**-**	**+**
DNA topoisomerase III (PSPPH_B0043)	**-**	**+**	**-**	**+**
T3SS helper protein HrpW1 (PSPPH_1264)	**+**	**-**	**-**	**-**
T3SS effector HopF3 (PSPPH_3498)	**+**	**-**	**-**	**-**
T3SS effector HopAA1 (PSPTO_1372)	**+**	**-**	**-**	**-**
T3SS effector AvrPto1 (PSPTO_4001)	**+**	**-**	**-**	**-**
T3SS chaperone protein SchF (PSPPH_3499)	**+**	**-**	**-**	**-**
Microcin biosynthesis (GenBank: ACXS01000133) *	**+**	**-**	**-**	**-**
Type VI secretion system (T6SS) (GenBank: ACXS01000079) *	**+**	**-**	**-**	**-**
Filamentous haemagglutinin-like protein (GenBank: ACXT01000416) *	**+**	**-**	**-**	**-**
Peptide methionine sulfoxide reductase (GenBank: ACXS01000236) *				
Putative shikimate kinase (PSPPH_A0133)	**+**	**+**	**-**	**+**
Short (61 a. a.) hypothetical protein (PSPPH_A0134)	**+**	**+**	**-**	**+**
Hypothetical protein (PSPPH_A0110)	**+**	**+**	**-**	**+**
Putative sulphotransferase (PSPPH_A0109)	**+**	**+**	**-**	**+**
Putative adenosylmethionine-8-amino-7-oxononanoate aminotransferase (PSPPH_A0108)	**+**	**+**	**-**	**+**
Putative SanC oxygenase (PSPPH_A0107)	**+**	**+**	**-**	**+**
Hypothetical protein (PSPPH_A0106)	**+**	**+**	**-**	**+**
PbsX-family transcription factor (PSPPH_B0022)	**+**	**+**	**-**	**+**
Fatty acid biosynthesis (GenBank: ACXT01000043)	**-**	**+**	**+**	**+**
Sucrose utilisation (PSPPH_5179 - PSPPH_5197)	**-**	**+**	**+**	**+**
Plasmid replication and conjugation (GenBank: GG700389)	**-**	**+**	**+**	**+**
Filamentous haemagglutinin-like protein (GenBank: ACXS01000449)	**-**	**+**	**+**	**+**
Iron uptake (GenBank: ACXT01000045)	**-**	**+**	**+**	**+**
Fatty-acid biosynthesis (Spro_2863 – Spro2869; GenBank: ACXT01000043)	**-**	**+**	**+**	**+**

+ = gene present.

- = gene absent.

*see Text S1 for full description.

**Figure 5:**
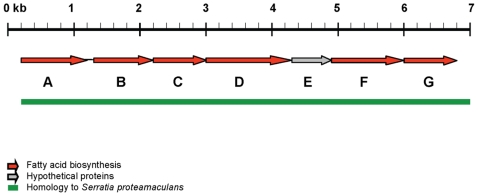
An E-*Pae*-encoded pathway for the biosynthesis of fatty acids. Shown is a cluster of genes in E-*Pae* implicated in fatty acid biosynthesis (indicated by the red arrows) with homology to *Serratia proteamaculans*, but which is absent in I-*Pae* and other *P. syringae* pathovars. The seven genes (A–G) occupy the entire 6.8 kb contig (GenBank: ACXT01000043). Full details of the predicted genes based on blastp searches are shown in Table 4.

E-*Pae* encoded seven predicted proteins which were not present in I-*Pae* and which had the highest amino acid sequence identities and synteny with a cluster of genes (Spro_2863-2869) from *Serratia proteamaculans* 568, an endophytic bacterium isolated from the roots of a woody host, *Populus trichocarpa* [27] (Figure 5, Table 4). This unique E-*Pae* gene cluster (0961-0967) also shared homology with the bacterial soft rot pathogen, *Pectobacterium carotovorum* subspecies *carotovorum* (synonym *Erwinina carotovora*) strain PC1 (PC1_4136-4142) but shared no nucleotide sequence similarity with other sequenced *P. syringae* pathovars. Although the function of this gene cluster has not been described for *S. proteamaculans* or *P. carotovorum* subspecies *carotovorum*, several of the genes are implicated in fatty acid biosynthesis (Table 4).

**Table 4:** Genes in E-*Pae* that may be involved in fatty acid biosynthesis.

ORF	E-*Pae* protein locus tag	Predicted function	Amino acid identity	*Serratia proteamaculans* locus tag
A	PSAESCULI2250_0961	β-ketoacyl-acyl carrier-protein synthase III	80%	Spro_2869
B	PSAESCULI2250_0962	NAD-dependent epimerases/dehydratase	72%	Spro_2868
C	PSAESCULI2250_0963	β-lactamase domain containing protein	77%	Spro_2867
D	PSAESCULI2250_0964	Putative adenylate forming enzyme	77%	Spro_2866
E	PSAESCULI2250_0965	Hypothetical protein	62%	Spro_2865
F	PSAESCULI2250_0966	Fatty acid hydroxylase	67%	Spro_2864
G	PSAESCULI2250_0967	Fatty acid desaturase	71%	Spro_2863

These proteins are encoded on a 6.8 kb contig (GenBank: ACXT01000043) as depicted in Figure 5.

**Figure 6:**
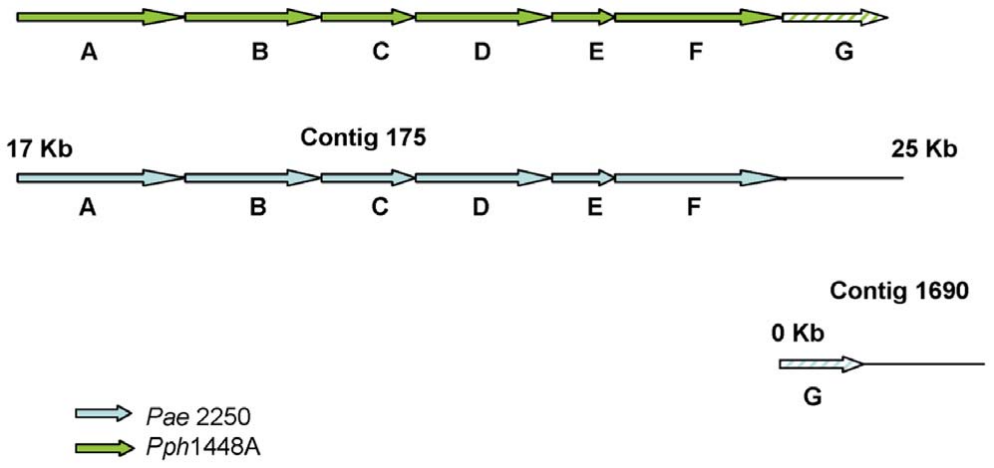
An E-*Pae*-encoded pathway for the utilization of sucrose. Shown is a cluster of genes in E-P*ae* which is implicated in the uptake and utilization of sucrose but which is not found in I-*Pae*. Details of the predicted genes based on blastp searches are shown in Table 5. This gene cluster is homologous to a region of *Pph* 448A (96–98% nucleotide sequence identity). In E-*Pae* the six principal genes (A–F) are on a 7.4 kb section of a 32.1 kb contig (GenBank: ACXT01000147.1) whereas the transcriptional regulator (G) is found at the beginning of a 12 kb contig (GenBank: ACXT01000532.1); (the first 67 codons are missing from the start of the contig). Genes in E-*Pae* are indicated by the blue arrows; genes in *Pph* 1448A are represented by the green arrows.

E-*Pae* harbored a cluster of genes predicted to be involved in sucrose uptake and utilization including genes for a putative sucrose porin and a sucrose (invertase) enzyme, SacA (Figure 6, Table 5) [28], [29]. These genes were conserved in *Pph* 1448A but were absent from I-*Pae*. Also present in E-*Pae* were several genes involved in iron sensing and transport, including iron and haemin ABC transporters, TonB-dependent outer-membrane siderophore receptors and iron-responsive regulators which were not present in I-*Pae*. For example, E-*Pae* genes 0996-0998 (GenBank: ACXT01000045) encoded a TonB-dependent receptor and two proteins resembling the ferric-dicitrate responsive regulatory system, FecIR.

**Table 5:** Genes in E-*Pae* that are likely to be involved in sucrose utilization (as depicted in Figure 6).

ORF Label in Figure 6	Protein locus tag	Predicted function
A	PSAESCULI2250_2446	Sucrose porin
B	PSAESCULI2250_2447	Sugar ABC transporter
C	PSAESCULI2250_2448	Sugar ABC transporter
D	PSAESCULI2250_2449	Sugar ABC transporter
E	PSAESCULI2250_2450	Sugar ABC transporter
F	PSAESCULI2250_2451	Sucrose-6-phosphate hydrolase
G	PSAESCULI2250_5084	LacI family sucrose transcriptional regulator

E-*Pae* and I-*Pae* each encoded a filamentous hemagglutinin (FHA)-like protein (E-*Pae* genes 3169 and 4454; I-*Pae* genes 3880-3883) recognizable by the presence of a haemagglutination activity domain (Pfam:PF05860). The FHA-like proteins in each strain had distinctly different sequences; in E-*Pae* the protein was very closely related to *Pto* DC3000 protein PSPTO_3229 and was conserved at the nucleotide sequence level in *Pta* 11528, but not in *Psy* B728, *Pph* 1448A or *Por 1-6*. In contrast, the gene encoding a FHA-like protein in I-*Pae* showed no detectable nucleotide sequence similarity to any previously sequenced genome. However, at the protein level this sequence most closely resembled FHA-like sequences from *Yersinia* spp. and *Pectobacterium* spp. (up to 61.5% identity over a 272 amino acid conserved region near the N terminus) (Figure S3). These FHA-like proteins may have a role in attachment to host cells, also of bacterial cells to each other in biofilm production, or possibly to insect vectors since haemagglutinin-like proteins were found to be associated with adhesion of *Xylella fastidiosa* to leafhopper vectors [30].

### Type III secretion systems in *Pae*

The type III secretion system (T3SS) plays a central role in pathogenicity of *P. syringae*. The genome of *Pph* 1448A encodes two distinct T3SS: the Hrp T3SS, which is responsible for delivering effectors into plant host cells, and a second system whose function is unknown [13]. Both T3SS gene clusters were conserved in *Pae* (Table S3). Since T3SS effectors play a key role in the ability of *P. syringae* to overcome plant host defences, we wanted to compare the effector repertoires of *Pae* strains with those of previously studied *P. syringae* strains. Both I-*Pae* and E-*Pae* contained genes encoding orthologues of AvrA1, AvrB4, AvrE1, AvrPto1, HopA2, HopAB1, HopAE1, HopAF1, HopAH2, HopAM1, HopAO1, HopAS1, HopD1, HopF1, HopG1, HopI1, HopM1, HopO1, HopF2, HopQ1, HopR1, HopT1, HopV1 and HopX1 (File S1).

### The “dispensable genome” of *Pae*

A bacterial genome consists of two compartments: a “core genome” containing genes conserved in all the strains of a given species, and a “dispensable genome” containing genes that are absent from one or more strains. Together, these two components make up the “pan-genome” [31]. Comparative studies of previously sequenced genomes [11]–[15], [32] have revealed a large pan-genome for the species *P. syringae*; up to 30% of the genome of a given strain is absent from strains of distantly related pathovars. However, there has been little investigation of the dynamics of the dispensable genome over very short phylogenetic distances, such as within a single pathovar.

Among the three E-*Pae* strains, only few genes had been gained or lost since their divergence from a common ancestor. E-*Pae* strain P6617 lacked close homologues of eight genes that were conserved among E-*Pae* strains 2250 and P6623, and *P. syringae* pv. *phaseolicola* (*Pph* 1448A) (Table 3). In *Pph* 1448A, these genes are located on the plasmids and so are likely also to be located on plasmids in *Pae*. Therefore gain or loss of whole plasmids might explain the variation in gene-content among E-*Pae* strains. Additionally, the presence of mobile genetic elements including Tn*3* family transposons and insertion sequence elements from several families (IS*3*, IS*5*, IS*21*, IS*66*, IS*91*, IS*111A*, IS*RSO5*) may also facilitate genomic variation in E-*Pae*.

We found that about 5% of either genome differed between I-*Pae* and E-*Pae*, thus comprising part of the dispensable genome for this pathovar. This degree of gene loss and gain is consistent with the greater nucleotide divergence between E-*Pae* and I-*Pae* (compared with that among E-*Pae strains*). Out of the 245 predicted genes in E-*Pae* that were absent from I-*Pae*, 170 were located on contigs that exhibited some nucleotide sequence similarity with plasmids in other bacterial genomes. These included genes involved in bacterial conjugation and plasmid transfer. For example, a 52.7 kb E-*Pae* scaffold (GenBank: GG700389) contains 31 predicted genes, including 14 conjugal transfer (*tra*) genes, that are homologous to genes located on plasmids from the tomato pathogen *Pto* DC3000. In addition, some of the genomic regions that differed between E-*Pae* and I-*Pae* were homologous to phage sequences (*e.g.* GenBank: GG700353 in E-*Pae* and GenBank: ACXS01000599 in I*-Pae*).

### Variation in plasmid content among *Pae* strains

**Figure 7:**
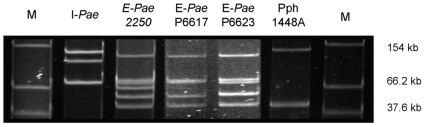
Plasmid profiles of *Pae*. Agarose gel electrophoresis was carried out as described by [33] to compare the number and size of native plasmids present within the genomes of each of the four *Pae* strains; *P. syringae* pv. *phaseolicola* strain 1448A was included for comparison. M represents marker plasmids from *Escherichia coli* strain 39R861 [60]. Note that E-*Pae* strains 2250 and P6623 have two similarly sized plasmids of *ca*. 70 kb.

Since plasmids might account for a significant part of the large-scale genomic differences among strains of *Pae*, we compared the number and size of native plasmids present within the genomes of each of the four *Pae* strains by agarose gel electrophoresis [33]. We found that all four *Pae* strains harbored native plasmids (Figure 7A). I-*Pae* had a different complement of plasmids (three plasmids of *ca*. 70, 100 and 120 kb in size) compared with E-*Pae* strains, all of which harboured 4–6 plasmids. The E-*Pae* strains also varied in their plasmid complement, with strain 2250 carrying a unique plasmid of *ca*. 66 kb and strain P6617 lacking another plasmid (*ca*. 70 kb) present in both 2250 and P6623.

## Discussion

We have exploited high-throughput sequencing technology to perform genome-wide surveys of genetic variation in *Pae*, the causative agent of bleeding canker of European horse chestnut. This has provided novel insights into the evolutionary origin of the pathogen and has revealed a suite of genes present in E-*Pae* which may facilitate its virulence and fast rate of spread on European horse chestnut. The *Pae*-specific pathways identified here are potentially highly important for the understanding of bacterial diseases of woody plants. It is clear that comparative genomics can quickly generate large amounts of genetic information on newly emerging plant diseases that will be valuable in development of strategies to combat future biosecurity threats posed by phytopathogens.

Since *Pae* is a newly emerging disease of unknown origin, we wanted to confirm the taxonomic placement of E-*Pae* strains causing the epidemic and determine the evolutionary relationships between *Pae* and other *P. syringae* pathovars. On the basis of seven house-keeping genes whose sequences are commonly used as phylogenetic markers [17], E-*Pae* strains recently isolated from the woody parts of diseased trees in Britain were identical to the I-*Pae* type-strain isolated from leaf tissues in India five decades ago. This close phylogenetic relationship is consistent with the classification of both I-*Pae* and E-*Pae* within the same pathovar of *P. syringae*. We also revealed that *Pae* belongs to a distinct clade of *P. syringae* genomospecies 2 pathovars that specialize on woody hosts. An interesting feature of this clade is that pathovar *mori*, which causes bacterial leaf spot of mulberry [34] and was isolated from leaves, lies outside a sub-group comprising *Pae* and pvs. *morsprunorum*, *savastanoi* and *myricae*, all of which cause cankers or galls in woody organs [35], [36]. Thus, this lineage within genomospecies 2 appears to have first colonized woody hosts, then adapted to infecting the woody parts of the host. These niche changes are likely to have required genetic adaptations, including the acquisition of new suites of genes and probably loss of redundant genes. I-*Pae*, however, is only known to infect leaves of Indian horse chestnut in its native region and we speculate that it may have only recently lost the ability to infect woody organs.

A number of economically important tree diseases are caused by *P. syringae*, including pvs. *syringae* and *morsprunorum* on stone fruit, *savastanoi* on olive and *avellanae* on hazelnut. Nonetheless, the virulence traits that enable infection of woody organs remain unknown [36], [37]. E-*Pae* is remarkably aggressive on European horse chestnut in causing extensive stem cankers that can kill large, mature trees within one to two seasons following infection. We therefore expected that E-*Pae* might possess a suite of genes required for pathogenesis in woody parts of the host. Using comparative genomics we were able to identify genomic regions present in *Pae* that share no sequence similarity to previously sequenced *P. syringae* genomes and which appear to code for traits potentially useful for fitness on a tree host. It remains to be seen whether any of these apparently *Pae*-specific genomic regions, discussed below, are also conserved in other *P. syringae* pathovars of woody hosts for which few sequence data are currently available.

Of particular significance for the aetiology of the disease epidemic on European horse chestnut are the *Pae-*encoded pathways for the degradation of plant-derived aromatic compounds such as lignin derivatives and other phenolics. These involve the catechol branch of the *β*-ketoadipate pathway and protocatechuate degradation via the protocatechuate 4,5-dioxygenase pathway. The *β*-ketoadipate pathway consists of two parallel branches for the catabolism of catechol and protocatechuate, derived from benzoate and 4-hydroxybenzoate, respectively, and plays a central role in the degradation of naturally occurring aromatic compounds derived from lignin and other plant components [22]. Other sequenced *P. syringae* pathovars on herbaceous hosts harbor genes that encode for the protocatechuate branch only [13]. This branch degrades derivatives of vanillate, an important intermediate metabolite in the microbial degradation of lignin-derived compounds [38]. The fact that *Pae* possesses genes that encode both the catechol and protocatechuate branches of the pathway implies that it has the additional ability to utilize unsubstituted lignin-related compounds such as cinnamate, as well as other plant-derived phenolic compounds including mandalate (2-hydroxy-2-phenylacetate) and phenol [22].

The protocatechuate 4,5-dioxygenase pathway, also apparently encoded by *Pae*, is a route for the degradation of protocatechuate that is currently not well understood [39]. The 4-hydroxybenzoate transporter protein encoded by *Pae* has been found to play a role in establishing the preferential degradation of benzoate via the catechol branch of the *β*-ketoadipate pathway in *P. putida* [40]. The beta-subunit of protocatechuate 4, 5-dioxygenase, also encoded by *Pae*, is known as *LigB* in *Sphingomonas paucimobilis*, which is a bacterium well characterised for its ability to utilize various types of lignin-derived biaryls [39]. This enzyme, which employs iron as a co-factor, is known to be involved in the degradation of protocatechuate from vanillate [38], as well as in the degradation of 3-*0*-methylgallate. This latter substrate is a metabolite of syringate, itself an important, lignin-derived monoaryl [41]. Protocatechuate 4,5-dioxygenase also has identity with the *ligZ* gene in *S. paucimobilis* that has a role in the biphenyl catabolic pathway of lignin degradation [39].

*Pae* is the first pathovar of *P. syringae* found to harbor these genes that encode the catechol and protocatechuate 4,5-dioxygenase pathways for degradation of aromatic compounds. These are pathways commonly associated with soil-dwelling bacterial species such as *P. putida* and *Acinetobacter* spp. known for their ability to break down a wide range of aromatic compounds including those derived from plants. It is possible that these pathways enable *Pae* to utilize as carbon sources aromatic substrates specifically derived from the tissues of woody plants. Other substrates may include phenols, coumarins and tannins laid down by tree hosts as structural defense responses to disease-causing organisms [41]. Microscopic observations of young branches of European horse chestnut naturally infected by *Pae* reveal that the bacterium causes cellular disruption in the cortex, phloem, and cambium [Steele *et al.*, unpublished]. In these *Pae-*infected branches the xylem can be plugged and discolored although xylem vessels are not apparently degraded. Future functional analyses through mutagenesis and complementation experiments will evaluate the precise role of these identified genes in infection of woody organs.

Iron is a major limiting nutrient in microbial growth, and pathways for the efficient uptake and utilization of iron are essential virulence factors in pathogenic bacteria [42]. *Pae* encoded a number of genes not yet found in other pathovars of *P*. *syringae* that are involved in iron acquisition, the most significant being a pathway for enterobactin siderophore biosynthesis. Enterobactin is the siderophore with the highest known affinity, primarily described in Enterobacteriaceae [42]. Although an ecological role for enterobactin has yet to be discovered, the presence of this gene cluster could confer upon *Pae* a considerable fitness advantage, particularly in an iron-limited environment.

Another mechanism in *Pae* that might be important to survival during host infection is the presence of the two genes that have a predicted function in nitric oxide metabolism. Both enzymes encoded by these genes have a role in the protection of bacteria from NO, which is an antimicrobial toxin produced by a host's immune response [23]. Neither gene has been found previously in *P. syringae*. Importantly, NO has been shown to play a key role in plant disease resistance by acting as a signal which induces plant genes to synthesise defense-related products [24]. Inhibitors of NO synthesis thereby compromise the plant's disease-resistance response and promote bacterial growth *in planta* [24]. *Pae* also encodes a novel toxin pathway not yet found in other *P. syringae* pathovars which appears to involve a CADD-type redox protein [26]. Although the function of such putative novel pathways cannot currently be predicted with certainty, it is possible that they function as toxins active against host plants, competing microbes, or insect vectors [43].

There were several genomic regions present in E-*Pae* which were absent from I-*Pae* and which may represent genetic adaptations specifically required for infection of the woody parts of *Aesculus*. These include a gene cluster with homology to *Serratia proteamaculans* 568 and *Pectobacterium carotovorum* subspecies *carotovorum*. The role of this cluster is unknown, but based on the predicted functions of the proteins encoded in E-*Pae* we suggest that it is a novel pathway for the biosynthesis of a long-chain fatty acid associated with the bacterial cell wall. Given that this E-*Pae* gene cluster is found in root-infecting bacteria but absent from other sequenced *P. syringae* pathovars, the encoded fatty acid could function as a permeability barrier, providing protection to bacterial cells in the harsh environments associated with soils or woody organs, similar to the role of mycolic acids in *Mycobacterium* species [44].

Our genomic comparison revealed the presence of sucrose utilization genes in E-*Pae*, but not in I-*Pae*. Sucrose is the predominant form of translocated carbohydrate within plants [45] and can represent over 95% of the dry weight of material translocated in the sieve tubes of the phloem [41]. Strains of E-*Pae* isolated from bleeding cankers on European horse chestnut cause lesions in the phloem of stems and branches, are isolated consistently from phloem tissue and may use the phloem as a conduit for spread within the tree [Steele et al., unpublished], unlike the leaf-infecting I-*Pae*. Therefore, an ability to utilize sucrose as a carbon source could be advantageous to growth of E-*Pae* within the stem and branches of European horse chestnut.

E-*Pae* and I-*Pae* encoded distinctly different filamentous hemagglutinin (FHA)-like proteins, which may be a reflection of their different mechanisms for infection. FHA are large beta-helical proteins, with the best-characterized example being the *Bordetella pertussis* FHA which appears to enable adhesion to eukaryotic host cells. During infection of mammals by *Bordetella*, FHA may also have additional functions such as immuno-suppression [46], [47] and host specificity [48]. In bacterial plant pathogens, FHA-like proteins appear to play a role in disease by facilitating adhesion between bacterial cells and plant host cells [49], [50] In *Xylella fastidiosa*, a vascular pathogen of grapevines, inactivation of the FHA-like protein (HxfA) led to hypervirulence which suggests that haemagglutinins mediate contact between bacterial cells, resulting in formation of colonies and biofilms within xylem vessels [51].

Some of the larger-scale genomic differences among the four *Pae* strains appeared to be associated with plasmids and all four strains varied in their plasmid complement. It is apparent, for example, that E-*Pae* has acquired a suite of conjugal transfer genes with similarity to *Pto* DC3000 plasmids which are absent from I-*Pae*. Since plasmids are inherently transferable from one bacterial cell to another, even crossing between species, they can allow bacteria to adapt to new environments, possibly resulting in changes in virulence and fitness through horizontal gene transfer [52]. Thus, plasmids are almost certainly an important factor enabling rapid evolutionary change in *Pae*. The observed variation among *Pae* strains in terms of plasmids, genes and genetic pathways is probably facilitated by the numerous phage and mobile genetic elements in their genomes [52], [53]. In fact, the abundance of insertion sequence (IS) elements in the genome of E-*Pae* was the reason why it was technically not feasible to take its sequence assembly to closure.

Through our comparative genome analysis we sought information on the evolution and likely origin of E-*Pae* on European horse chestnut. The degree of divergence between E-*Pae* and I-*Pae* was found to be very low (about 0.05% across aligned genomic sequences) indicating that they shared a recent common ancestor. However, the near identity among the three E-*Pae* strains from diverse locations in Britain (only one or two nucleotide differences across 3 Mbp) indicates that their common ancestry is much more recent, and consistent with a single introduction within the last few years. This serves to highlight the environmental risks posed by the spread of exotic plant pathogens into new geographical locations. In contrast to our findings for strains from Britain, REP-PCR profiles have been reported to vary among approximately 50 *Pae* strains isolated from diseased European horse chestnut in Belgium [54]. Since *Pseudomonas* genomes do not contain copies of the repetitive sequences from which the REP-PCR primers were designed, this profiling technique is analogous to using arbitrary or random primers [55], and it is not known whether the sequences amplified were chromosomal or from plasmids. Thus, it is as yet unclear whether E-*Pae* strains from outside Britain exhibit greater genetic variation; if they do it would indicate that they have a longer history of divergence. It also remains uncertain whether E-*Pae* originates from India. More information on the geographic origin and routes of spread of E-*Pae* could be elucidated by phylogenetic analyses of a broad range of *Pae* strains from Europe, comparing the genetic variability of these strains with newly collected *Pae* strains from Indian horse chestnut in India.

This study demonstrates the value of genome-wide sequence data for surveying intra-pathovar genetic variation among *phytopathogenic* strains that were indistinguishable using existing molecular markers. The comparative genomics approach has enabled us to identify SNPs and other variable regions in *Pae* that offer candidate molecular markers for large scale phylogenetic analyses. Also, the presence of common regions in the genomes of geographically distinct E-*Pae* strains suggests that these regions may be highly conserved and may thus provide appropriate loci for the development of diagnostic markers that can differentiate E-*Pae* and I-*Pae*. Such tools are needed to support phytosanitary measures aimed at preventing the introduction of *Pae* to new geographical areas, such as North America where it could present a serious threat to various native *Aesculus* spp.

## Materials and Methods

### Bacterial strains

E-*Pae* strain 2250 was isolated from necrotic phloem in the stem of a diseased horse chestnut near Pitlochry, Perthshire, Scotland, in 2008. E-*Pae* strain P6617 was isolated from a diseased horse chestnut in Glasgow, Scotland in 2006, and E-*Pae* strain P6623 was isolated from a diseased horse chestnut near Farnham, Surrey, England in 2006. Prior to sequencing, the pathogenicity of E-*Pae* 2250 was confirmed by inoculating a cell suspension onto wounded horse chestnut shoots and observing subsequent development of lesions. I-*Pae* (NCPPB3681; also known as 0893_23 in the USA, D. Cooksey, Pers. Comm.) was isolated from a leaf lesion on Indian horse chestnut in 1969 in a temperate region of Northern India [8].

For E-*Pae*, initial isolations were made on nutrient agar amended with 5% w/v sucrose, crystal violet (2 mg/L) and Actidione (cycloheximide) (50 ng/L), to inhibit fungal growth and incubated at room temperature. Growth was visualized under UV light for blue fluorescence, and, if positive, fluorescent isolated bacterial colonies were subsequently streaked on to King's medium B [56]. Gram testing was done by adding a drop of 3% w/v aqueous KOH to a sub-sample of the colony on a microscope slide and confirming the presence of Gram negative isolates by an observed increase in viscosity. Isolates were stored at -80°C in Protect Bacterial Preservers (Technical Service Consultants Limited, Lancs, UK). To identify the strains, DNA was extracted and PCR carried out using the primer pair gyrB-F and gyrB-R [17]. The PCR product was sequenced and aligned with other bacterial gyrase B gene sequences available in GenBank and identified as *Pae* based on 100% similarity with a 470 bp gyrase B gene fragment of *P. syringae* pv. *aesculi* strain 0893-23 (DQ072677; I-*Pae*) isolated from *A. indica* in India [9].

### Library preparation and Illumina sequencing

DNA was extracted from I-*Pae* and E-*Pae* strains P6617 and P6623 grown in nutrient broth using the Puregene Genomic DNA Purification Kit (Gentra Systems, Inc., Minneapolis, USA) according to manufacturer's instructions. For E-*Pae* strain 2250, DNA was extracted using the DNeasy Plant Mini Kit (Qiagen). A library for Illumina Paired-End sequencing was prepared from 5 mg DNA using a Paired-End DNA Sample Prep Kit (Pe-102-1001, Illumina, Inc., Cambridge, UK). Sample DNA concentration was measured using Nanodrop and concentrations were equalized amongst the samples. Finally, DNA integrity was assessed using agarose gel electrophoresis. DNA was fragmented by nebulisation for 6 min at a pressure of 32 psi. For end-repair and phosphorylation, sheared DNA was purified using QIAquick Nucleotide Removal Kit (Qiagen, Hilden, Germany). The end repaired DNA was A-tailed and adaptors were ligated according to manufacturer's instructions. Size fractionation and purification of ligation products were performed using a 5% polyacrylamide gel run in TBE at 180 V for 120 min. Gel slices were cut containing DNA in the 10 to 500 bp range. DNA was then extracted using 0.3 M sodium acetate and 2 mM EDTA [pH 8.0] followed by ethanol precipitation. Using 18 PCR cycles with primer PE1.0 and PE2.0 supplied by Illumina, 5′ adaptor extension and enrichment of the library were performed. The library was finally purified using a QIAquick PCR Purification Kit and adjusted to a concentration of 10 nM in 0.1% Tween. The stock was kept at −20°C until used. We generated 9.66 million, 11.13 million, 10.56 million and 8.86 million usable pairs of 36-nucleotide reads from genomic DNA of the four strains of *Pae* using the Genome Analyzer II (Illumina). This represents approximately 116, 133, 127, 106 X coverage of a 6 Mb genome (the expected size, based on previously sequenced *P. syringae* pathovars).

### Whole-genome assembly

We assembled the E-*Pae* strain 2250 and I-*Pae* Illumina datasets *de novo* (*i.e.* without using a reference genome) using Velvet 0.7.48 [57]. Note that these sequence datasets probably also include sequence reads that originate from plasmids as well as from the chromosome. For assembly of the 17.7 million E-*Pae* Illumina paired reads, we used Velvet hash-length  = 27 and coverage cut-off  = 5. For assembly of the 19.3 million I-*Pae* Illumina paired reads, we used Velvet hash-length  = 21 and coverage cut-off  = 4. The different parameter values used for each strain yielded the best balance of contiguity and accuracy for their respective datasets. We used the FgenesB pipeline to predict protein-coding genes. Quality control procedures for genome assemblies are described in Text S1. The genome assemblies have been deposited in GenBank with accession numbers ACXS00000000 (I-*Pae*) and ACXT00000000 (E-*Pae*).

### Alignment of Illumina reads against a reference sequence

We used the Mapping with Alignment Qualities (MAQ) package [58] version 0.6.8.

### Detection of genes present and absent based on alignment to a reference sequence

We aligned the complete set of Illumina sequence reads for E*-Pae* 2250 and I-*Pae* against their respective *de novo* genome assemblies using MAQ (using the default parameter settings). For E-*Pae*, 16883015 /17726652 (95%) of the sequence reads aligned and for I-*Pae*, 8074128/19322678 (93.5%) of the sequence reads aligned. The unassembled portion of each genome was largely comprised of repeated sequences, such as tRNA and rRNA genes and transposable elements. The Illumina sequence reads are likely to represent the entire genomes of E-*Pae* and I-*Pae g*iven their high depth of coverage. This assumption is supported by a recent study of the *de novo* assembly of the *Psy* B728a genome using short sequence reads [59]. Therefore, we based our inferences of gene-presence/absence in E-*Pae* and I-*Pae* on alignments of the unassembled sequence reads as well as comparison of the *de novo* genome assemblies against each other and against previously published reference genome sequences.

After aligning Illumina reads against the reference sequence of *Pph* 1448A, we would expect that genes that are conserved between *Pph* 1448A and *Pae* should be covered by Illumina reads over most or all of their length. If this proposition is true, then we can identify those *Pph* 1448A genes that are not conserved in *Pae* on the basis of their lack of coverage by Illumina reads. We tested the reliability of this approach using a set of 683 *Pph* 1448A genes that are highly conserved in *Pto* DC3000 and *Psy* B728a (and therefore we expect that most are also conserved in E-*Pae*). These genes are listed in File S1. Of these 683 genes, 679 (99.41%) were covered by E-*Pae* Illumina reads over at least 85% of their length. Only one gene was less than 50% covered by E-*Pae* Illumina reads. Similarly, 677 (99.12%) of the highly conserved genes were at least 85% covered by I-*Pae* Illumina reads.

### Identification of single nucleotide polymorphisms (SNPs) from Illumina data

We used MAQ alignments of Illumina sequence reads versus the E-*Pae* genome assembly to detect SNPs. We only considered the 2,698,682 nucleotides in the E-*Pae* assembly for which there was at least 40X depth of coverage by Illumina reads from each of the four *Pae* datasets and there was at least 95% consensus between the aligned reads. We considered a SNP to be present at a given site if at least 95% of the aligned reads at that site consistently call a different nucleotide from that in the reference sequence. The remainder of the genome was considered to be ambiguous, and we made no attempt to determine whether SNPs were present or absent there.

### Determining the phylogenetic position of *Pae* within *P. syringae*

To investigate the position of *Pae* within the evolutionary radiation of *P. syringae* pathovars, we used the partial sequences of seven housekeeping genes (*acnB*, *fruK*, *gapA*, *gltA*, *gyrB*, *pgi* and *rpoD*) analyzed in [17]. We added the sequences from the four *Pae* isolates, and those from *P. syringae* pv. *phaseolicola* 1448A [13] and *P. syringae* pv. *tabaci* 11528 [14], to those from the 60 strains examined by [17]. The concatenated sequences yielded an alignment with 3,129 sites that could be compared among all strains. Where there were identical sequences from multiple strains assigned to the same pathovar, only one sequence was retained. The phylogenetic relationships among these sequences were estimated using the Bayesian Markov chain Monte Carlo method implemented in MrBayes v3.1.2 [60], run for 2,000,000 generations with a burn-in time of 500,000. The general time reversible model of nucleotide substitution was used, with gamma-distributed among-site rate variation, and a proportion of invariant sites. The tree was rooted according to [17].

### Analysis of plasmid sizes

Agarose gel electrophoresis was carried out as described by [33] to compare the number and size of native plasmids present within the genomes of each of the four *Pae* strains; *P. syringae* pv. *phaseolicola* strain 1448A was included for comparison. M represents marker plasmids from *Escherichia coli* strain 39R861 [61].

### Sequence similarity searches

BLAST [62] using a threshold of 1e-10 was used for sequence similarity searches. For Pfam searches, the Pfam ‘gathering thresholds’ was used as determined by the Pfam annotators.

## Supporting Information

Table S1 Conservation of predicted Type VI Secretion System (T6SS) components in E- Pae and I-Pae.
(0.04 MB DOC)

Table S2 Putative Type VI Secretion system substrates in I-Pae and their orthologues in E-Pae.
(0.03 MB DOC)

Table S3 Repertoires of type III secretion system (T3SS) effectors in E-Pae and I-Pae.
(0.05 MB DOC)

Figure S1 Pae genes implicated in nitric oxide metabolism that are not conserved in previously sequenced P. syringae genomes. Shown is a 3 kb contig on the E-Pae genome with positions and FgenesB automatic gene predictions and annotations. Regions of sequence identity (based on blastn [16] searches with a significance threshold of 1e-10) to I-Pae and to previously sequenced P. syringae genomes are indicated by horizontal bars.
(0.09 MB PPT)

Figure S2 I-Pae encodes a microcin biosynthesis pathway that is absent from E-Pae and from previously sequenced P. syringae genomes. Shown is a 7 kb contig on the I-Pae genome with positions and FgenesB automatic gene predictions and annotations. Regions of sequence identity (based on blastn [16] searches with a significance threshold of 1e-10) to E-Pae and to previously sequenced P. syringae genomes are indicated by horizontal bars. Full details of the predicted genes are described in SUPPORTING INFORMATION.
(0.10 MB PPT)

Figure S3 E-Pae and I-Pae encode highly divergent filamentous hemagglutinin-like (FHA) proteins. We used MAFFT [17] to align the predicted Pae FHA protein sequences against similar proteins recovered from the NCBI Proteins database via blastp searches. We generated a phylogenetic tree using the Neighbour Joining method implemented by Quicktree [18].
(0.13 MB PPT)

File S1 Set of 683 Pph 1448A genes that are highly conserved in Pto DC3000 and Psy B728a.
(0.09 MB DOC)

Text S1 Text for supporting information.
(0.04 MB DOC)
