# Supplementary material for: Comparative Genome Analysis Provides Insights into the Evolution and Adaptation of Pseudomonas syringae pv. aesculi on Aesculus hippocastanum
Source: PLoS One. 2010 Apr 19;5(4):e10224. doi: 10.1371/journal.pone.0010224 (PMC2856684; doi:10.1371/journal.pone.0010224)
Supplement: Table S1 — Conservation of predicted Type VI Secretion System (T6SS) components in E- Pae and I-Pae. (0.04 MB DOC) [file pone.0010224.s001.doc]

| **Homologue in previously sequenced genome** | **T6SS effector** | **Sequence features** | **I-*Pae* predicted gene and genomic location (on Genbank accession)** | **E-*Pae* predicted gene and genomic location (on Genbank accession)** |
| --- | --- | --- | --- | --- |
| PSPTO_2538 | VgrG-2 | PF04524 (DUF586) | PSAESCULI_4731 (ACXS01000593: 7091-9178 ) | PSAESCULI2250_2014 (ACXT01000110: 788-3553); PSAESCULI2250_5243 (ACXT01000612: 2345-4432) |
| PSPTO_2546 | VasA | PF05947 (DUF879) | PSAESCULI_0788 (ACXS01000079: 17978-19627) | Absent |
| PSPTO_2547 | VasB | PF06996 (DUF1305) | PSAESCULI_0787 (ACXS01000079: 16787-17806) | Absent |
| PSPTO_2548 | ClpV | PF10431 (ClpB) | PSAESCULI_0786 (ACXS01000079: 14196-16790) | Absent |
| PSPTO_2549 | VasH | PF00158 (Sigma54 activator) PF02954 (HTH 8) | PSAESCULI_0785 (ACXS01000079: 13253-14209) | Absent |
| PSPTO_2550 |  |  | (ACXS01000079: 13203-13069) | Absent |
| PSPTO_2551 |  | Predicted lipoprotein | PSAESCULI_0784 (ACXS01000079.1: 12431-12877) | Absent |
| PSPTO_2552 | VasE | PF05936 (DUF876) | PSAESCULI_0783 (ACXS01000079: 11103-12434) | Absent |
| PSPTO_2553 | VasF | PF09850 (DUF2077) | PSAESCULI_0782 (ACXS01000079: 10231-11094) | Absent |
| PSPTO_2554 | VasK | PF06744 (DUF1215) | PSAESCULI_0781 (ACXS01000079: 6714-10217) | PSAESCULI2250_0455 (ACXT01000018: 2-2650) |
| PSPTO_5434 | VasJ/VasL | PF06812 (ImpA) | PSAESCULI_3407 (ACXS01000357: 1309-2859) | Absent |
|  |  |  |  |  |
| PSPPH_0131 | VasA | PF05947 (DUF879) | PSAESCULI_0984 (ACXS01000097: 6760-8595) | PSAESCULI2250_3097 (ACXT01000216: 1836-3671) |
| PSPPH_0130 | VasB | PF06996 (DUF1305) | PSAESCULI_0983 (ACXS01000097: 5726-6796) | PSAESCULI2250_3096 (ACXT01000216: 802-1872) |
| PSPPH_0129 | ClpV | PF10431 (ClpB) | PSAESCULI_0982 (ACXS01000097: 3126-5729) | Absent |
| PSPPH_0128 |  | Predicted lipoprotein | PSAESCULI_0981 (ACXS01000097: 2336-3046) | Absent |
| PSPPH_0127 | VasE | PF05936 (DUF876) | PSAESCULI_0980 (ACXS01000097: 996-2339) | Absent |
| PSPPH_0126 | VasF | PF09850 (DUF2077) | PSAESCULI_0979 (ACXS01000097: 286-999) | Absent |
| PSPPH_0125 | VasK | PF06744 (DUF1215) | PSAESCULI_0978 (ACXS01000097: 4-258) | Absent |
| PSPPH_0124 |  | PF09867 (DUF2094) | PSAESCULI_2884 (ACXS01000260: 3701-4618) | Absent |
| PSPPH_0123 |  | PF00691 (OmpA) | PSAESCULI_1774 (ACXS01000163: 4583-6547) | PSAESCULI2250_1813 (ACXT01000093: 29-1972) |
| PSPPH_0122 | Hcp |  | PSAESCULI_1773 (ACXS01000163: 3917-4435) | PSAESCULI2250_1814 (ACXT01000093: 2120-2638) |
| PSPPH_0121 | VasJ/VasL | PF06812 (ImpA) | PSAESCULI_1772 (ACXS01000163: 3429-3812) | PSAESCULI2250_1815 (ACXT01000093: 2743-3126) |
