# Supplementary material for: Comparative Genome Analysis Provides Insights into the Evolution and Adaptation of Pseudomonas syringae pv. aesculi on Aesculus hippocastanum
Source: PLoS One. 2010 Apr 19;5(4):e10224. doi: 10.1371/journal.pone.0010224 (PMC2856684; doi:10.1371/journal.pone.0010224)
Supplement: Table S2 — Putative Type VI Secretion system substrates in I-Pae and their orthologues in E-Pae. (0.03 MB DOC) [file pone.0010224.s002.doc]

| **I-*Pae* gene and genomic location (on Genbank accession)** | **Orthologue in E-*Pae*** | **Comment** |
| --- | --- | --- |
| PSAESCULI_1773 (ACXS01000163: 3917-4435) | PSAESCULI2250_1814 (ACXT01000093: 2120-2638) | Hcp. Protein sequence 30% identical to UniProt: Q9I747 (HCP1_PSEAE), which is actively secreted during chronic infection of cystic fibrosis patients [15]. 69% identical to Csal_2258 from *Chromohalobacter salexigens*. |
| PSAESCULI_1784 (ACXS01000165: 989-1477) PSAESCULI_2406 (ACXS01000214: 1365-1853) | PSAESCULI2250_1689 (ACXT01000087: 34253-34741) | Hcp. Protein sequence nearly identical to UniProt: Q4LBF6, from *Pseudomonas syringae* pv. *apii* |
| PSAESCULI_3406 (ACXS01000357: 189-707) | Absent | Hcp. Protein sequence is 96% identical to ZP_05642093 from *Pta* 11528. |
| PSAESCULI_5059 (ACXS01000645: 39347-39835) | PSAESCULI2250_2171 (ACXT01000120: 28704-29192) | Hcp. Protein sequence 31% identical to UniProt: Q9I747 (HCP1_PSEAE), which is actively secreted during chronic infection of cystic fibrosis patients [15]. 96% identical to Psyr_4039 frpm *Psy* B728a. |
| PSAESCULI_0538 (ACXS01000053: 445-750) | PSAESCULI2250_2467 (ACXT01000151: 451-756) | Hcp. Protein sequence 91% identical to PSPPH_4224 from *Pph* 1448A |
| PSAESCULI_0539 (ACXS01000053: 750-947) | PSAESCULI2250_2468 (ACXT01000151: 756-953) | Hcp. Protein sequence 91% identical to PSPPH_4224 from *Pph* 1448A |
| PSAESCULI_4731 (ACXS01000593: 7091-9178) | PSAESCULI2250_2014 (ACXT01000110: 788-3553) PSAESCULI2250_5243 (ACXT01000612: 2345-4432) | VgrG. 71% identical to PSPTO_2538 from Pto DC3000 |
