# Supplementary material for: Comparative Genome Analysis Provides Insights into the Evolution and Adaptation of Pseudomonas syringae pv. aesculi on Aesculus hippocastanum
Source: PLoS One. 2010 Apr 19;5(4):e10224. doi: 10.1371/journal.pone.0010224 (PMC2856684; doi:10.1371/journal.pone.0010224)
Supplement: Table S3 — Repertoires of type III secretion system (T3SS) effectors in E-Pae and I-Pae. (0.05 MB DOC) [file pone.0010224.s003.doc]

| **Hop name** | **Homologue in I-*Pae*** | **Homologue in E-Pae 2250** |
| --- | --- | --- |
| **AvrA1** (GenBank: M15194) | PSAESCULI_3150 (ACXS01000300: 228-2948) shares 96% identity over 907 of 907 a.a. | PSAESCULI2250_3532 (ACXT01000282: 440-3160) shares 97% identity over 907 of 907 a.a. |
| **AvrB4** (GenBank: AAX12108) | PSAESCULI_4478 (ACXS01000551: 32-994) shares 51% identity over 322 of 323 a.a. | PSAESCULI2250_4117 (ACXT01000363: 38-1000) shares (GenBank: AAX12108) 51% identity over 322 of 323 a.a. |
| **AvrE1** (GenBank: AF458405) | PSAESCULI_0179 (ACXS01000011: 26374-31518) shares 73% identity over 1150 of 1160 a.a. | PSAESCULI2250_1526 (ACXT01000080: 3531-8675) shares 73% identity over 1150 of 1160 a.a. |
| **AvrPto1** (GenBank: AAY39946) | PSAESCULI_4971 (ACXS01000633: 746-1222) shares 50% identity over 148 of 163 a.a. | PSAESCULI2250_4632 (ACXT01000451: 17-493) shares 50% identity over 148 of 163 a.a. |
| **HopA2** (GenBank: AAP23114) | PSAESCULI_2715 (ACXS01000238: 26946-28040) shares 92% identity over 364 of 382 a.a. | PSAESCULI2250_3000 (ACXT01000201: 8874-9968) shares 92% identity over 364 of 382 a.a. |
| **HopAB1** (GenBank: AJ439728) | PSAESCULI_0590 (ACXS01000056: 336-1190) shares 98% identity over 284 of 523 a.a.  **Truncated due to low-complexity sequence** | PSAESCULI2250_5174 (ACXT01000572: 909-1763) shares 98% identity over 284 of 523 a.a. **Truncated due to low-complexity sequence** |
| **HopAE1** (GenBank: AAZ36433) | PSAESCULI_4863 (ACXS01000616: 7883-10621) shares 98% identity over 912 of 912 a.a. | PSAESCULI2250_4032 (ACXT01000353: 7889-10627) shares 98% identity over 912 of 912 a.a. |
| **HopAF1** (GenBank: AAZ34270) | PSAESCULI_3154 (ACXS01000301: 1463-1873) shares 98% identity over 136 of 284 a.a.  **Truncated due to low-complexity sequence** | PSAESCULI2250_0439 (ACXT01000016: 331-1185) shares 99% identity over 284 of 284 a.a. |
| **HopAH2** (GenBank: AAZ34780.1) | PSAESCULI_2094 (ACXS01000182: 9766-11016) shares 98% identity over 416 of 416 a.a. | PSAESCULI2250_4809 (ACXT01000510: 17859-19109) shares 98% identity over 416 of 416 a.a. |
| **HopAM1** (GenBank: X84843) | PSAESCULI_2201 (ACXS01000193: 3-870) shares 89% identity over 271 of 276 a.a. | PSAESCULI2250_3416 (ACXT01000271: 11-844) shares 89% identity over 275 of 276 a.a. |
| **HopAO1** (GenBank: AAO58160) | PSAESCULI_4380 (ACXS01000523: 160-1515) shares 88% identity over 453 of 468 a.a. | PSAESCULI2250_4979 (ACXT01000521: 3148-4503) shares 88% identity over 453 of 468 a.a. |
| **HopAS1** (GenBank: AAZ37064) | PSAESCULI_1301 (ACXS01000123: 23659-27744) shares 98% identity over 1361 of 1361 a.a. | PSAESCULI2250_4549 (ACXT01000435: 5992-10077) shares 98% identity over 1361 of 1361 a.a. |
| **HopD1** (GenBank: EEB56626) | PSAESCULI_3055 (ACXS01000279: 1570-2910) shares 98% identity over 445 of 698 a.a. **FgenesB mis-predicts the N-terminal start codon, but the full length gene is present in the DNA sequence.** | PSAESCULI2250_3296 (ACXT01000257: 2112-3431) shares 98% identity over 439 of 698 a.a. **FgenesB mis-predicts the N-terminal start codon, but the full length gene is present in the DNA sequence.** |
| **HopF1** (GenBank: CAM12736) | PSAESCULI_5499 (ACXS01000769: 309-926) shares 56% identity over 203 of 204 a.a. | PSAESCULI2250_5154 (ACXT01000565: 843-1460) shares 56% identity over 203 of 204 a.a. |
| **HopG1** (GenBank: AAZ34904) | PSAESCULI_4181 (ACXS01000493: 388-1860) shares 53% identity over 496 of 515 a.a. | PSAESCULI2250_4419 (ACXT01000410: 394-1866) shares 53% identity over 496 of 515 a.a. |
| **HopI1** (GenBank: AAZ33342) | PSAESCULI_2360 (ACXS01000208: 31353-32363) shares 94% identity over 336 of 336 a. a. | PSAESCULI2250_2302 (ACXT01000132: 93-1103) shares 94% identity over 336 of 336 a. a. |
| **HopM1** (GenBank: AAY36240  ) | PSAESCULI_0180 (ACXS01000011: 31757-33880) shares 68% identity over 516 of 718 a.a. | PSAESCULI2250_1525 (ACXT01000080: 1007-3292) shares 67% identity over 624 of 718 a.a. |
| **HopO1** (GenBank: AF458392) | PSAESCULI_0792 (ACXS01000080: 840-1691) shares 99% identity over 283 of 283 a.a. | PSAESCULI2250_2457 (ACXT01000148: 873-1724) shares 99% identity over 283 of 283 a.a. |
| **HopF2** (GenBank: AAO54046) | PSAESCULI_5499 (ACXS01000769: 309-926) shares 54% identity over 203 of 204 a. a. | PSAESCULI2250_5154 (ACXT01000565: 843-1460) shares 54% identity over 203 of 204 a. a. |
| **HopQ1** (GenBank: AAZ37975) | PSAESCULI_3054 (ACXS01000279: 111-1454) shares 99% identity over 447 of 447 a.a. | PSAESCULI2250_3297 (ACXT01000257: 3547-4458) shares 99% identity over 303 of 447 a.a. **On the edge of contig.** |
| **HopR1** (GenBank: AAZ37024) | PSAESCULI_3530 (ACXS01000384: 5434-10560) shares 99% identity over 1708 of 1959 a.a.; PSAESCULI_3531 (ACXS01000385: 3-684) shares 99% identity over 227 of 1959 a.a. **Gene is split into two ORFs in the assembly.** | PSAESCULI2250_2455 (ACXT01000147: 25663-31542) shares 99% identity over 1959 of 1959 a.a. |
| **HopT1** (GenBank: AF458399) | PSAESCULI_0793 (ACXS01000080: 1697-2872) shares 98% identity over 379 of 379 a.a. | PSAESCULI2250_2458 (ACXT01000148: 1730-2905) shares 98% identity over 379 of 379 a.a. |
| **HopV1** (GenBank: AAZ34233) | PSAESCULI_3013 (ACXS01000273: 15911-16885) shares 99% identity over 324 of 595 a.a. **FgenesB mis-predicts the N-terminal start codon, but the full length gene is present in the DNA sequence.** | PSAESCULI2250_1051 (ACXT01000048: 8551-9525) shares 99% identity over 324 of 595 a.a. **FgenesB mis-predicts the N-terminal start codon, but the full length gene is present in the DNA sequence.** |
| **HopX1** (GenBank: AAP23110) | PSAESCULI_5572 (ACXS01000804: 3823-4692) shares 71% identity over 287 of 380 a.a. | PSAESCULI2250_4130 (ACXT01000375: 4035-4904) shares 71% identity over 287 of 380 a.a. |
