# Supplementary figures and images for: Comparative Genome Analysis Provides Insights into the Evolution and Adaptation of Pseudomonas syringae pv. aesculi on Aesculus hippocastanum
Source: PLoS One. 2010 Apr 19;5(4):e10224. doi: 10.1371/journal.pone.0010224 (PMC2856684; doi:10.1371/journal.pone.0010224)

## Slide 1
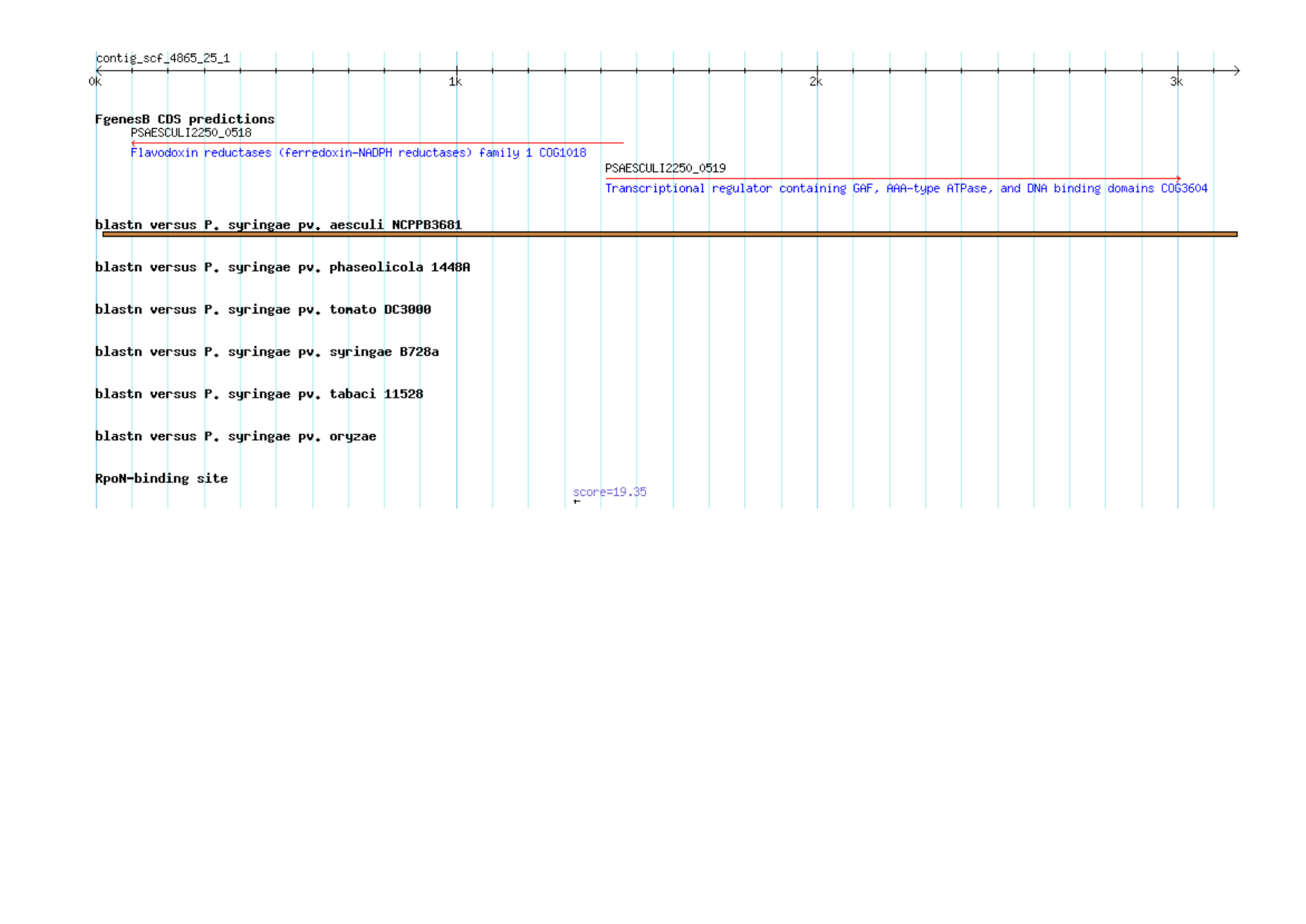

Supplement: Figure S1 — Pae genes implicated in nitric oxide metabolism that are not conserved in previously sequenced P. syringae genomes. Shown is a 3 kb contig on the E-Pae genome with positions and FgenesB automatic gene predictions and annotations. Regions of sequence identity (based on blastn [16] searches with a significance threshold of 1e-10) to I-Pae and to previously sequenced P. syringae genomes are indicated by horizontal bars. (0.09 MB PPT) [file pone.0010224.s004.ppt]

## Slide 1
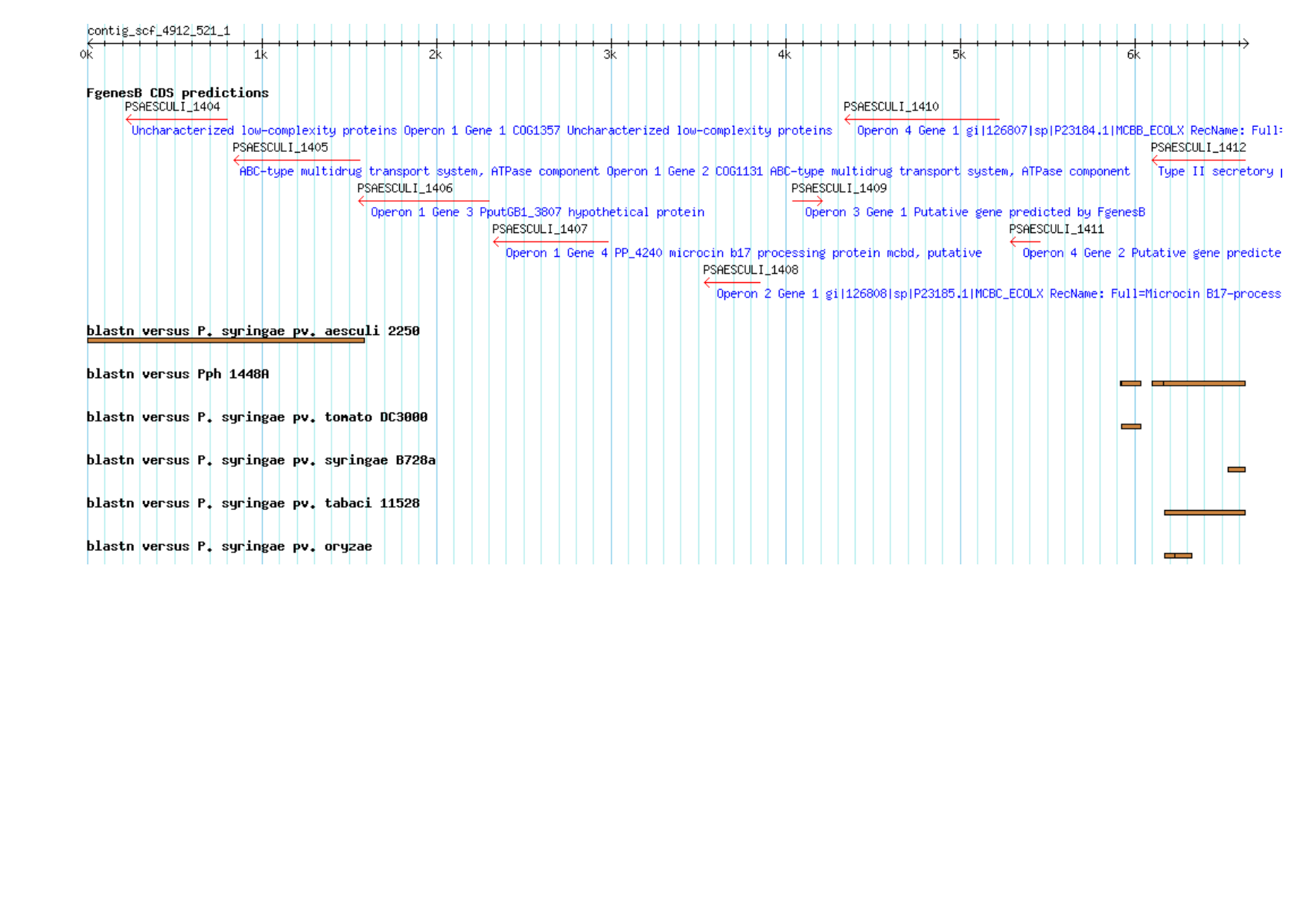

Supplement: Figure S2 — I-Pae encodes a microcin biosynthesis pathway that is absent from E-Pae and from previously sequenced P. syringae genomes. Shown is a 7 kb contig on the I-Pae genome with positions and FgenesB automatic gene predictions and annotations. Regions of sequence identity (based on blastn [16] searches with a significance threshold of 1e-10) to E-Pae and to previously sequenced P. syringae genomes are indicated by horizontal bars. Full details of the predicted genes are described in SUPPORTING INFORMATION. (0.10 MB PPT) [file pone.0010224.s005.ppt]

## Slide 1
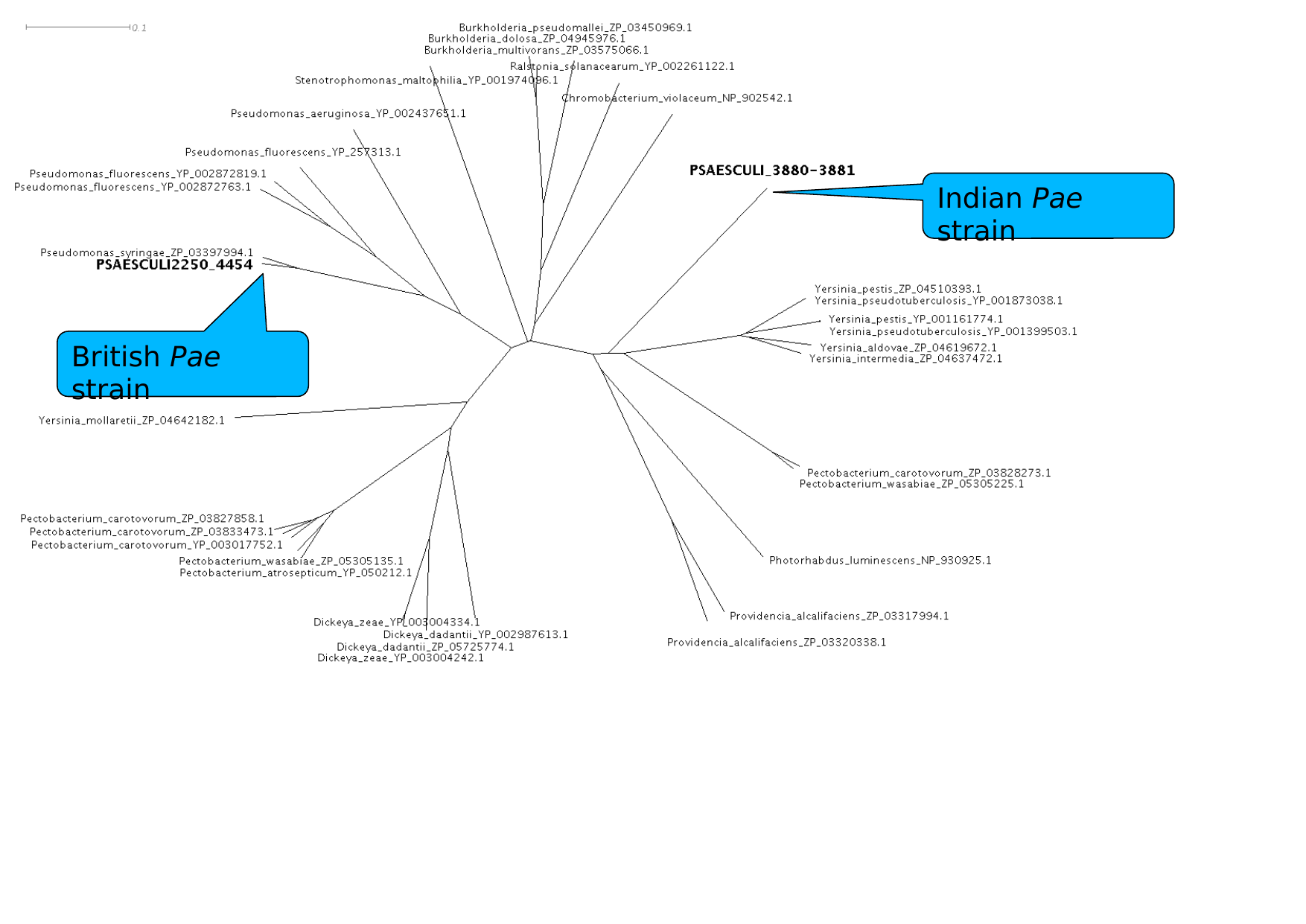

Indian Pae strain
British Pae strain

Supplement: Figure S3 — E-Pae and I-Pae encode highly divergent filamentous hemagglutinin-like (FHA) proteins. We used MAFFT [17] to align the predicted Pae FHA protein sequences against similar proteins recovered from the NCBI Proteins database via blastp searches. We generated a phylogenetic tree using the Neighbour Joining method implemented by Quicktree [18]. (0.13 MB PPT) [file pone.0010224.s006.ppt]
