# Supplementary material for: Comparative Genome Analysis Provides Insights into the Evolution and Adaptation of Pseudomonas syringae pv. aesculi on Aesculus hippocastanum
Source: PLoS One. 2010 Apr 19;5(4):e10224. doi: 10.1371/journal.pone.0010224 (PMC2856684; doi:10.1371/journal.pone.0010224)
Supplement: File S1 — Set of 683 Pph 1448A genes that are highly conserved in Pto DC3000 and Psy B728a. (0.09 MB DOC) [file pone.0010224.s007.doc]

>PSPPH_0003 recombination protein F

>PSPPH_0004 DNA gyrase subunit B

>PSPPH_0011 glycyl-tRNA synthetase subunit beta

>PSPPH_0012 glycyl-tRNA synthetase subunit alpha

>PSPPH_0013 DNA-3-methyladenine glycosidase I

>PSPPH_0014 lipid A biosynthesis lauroyl acyltransferase

>PSPPH_0020 methionyl-tRNA formyltransferase

>PSPPH_0024 SUA5/yciO/yrdC family domain protein

>PSPPH_0026 coproporphyrinogen III oxidase

>PSPPH_0030 choline sulfatase

>PSPPH_0036 tryptophan synthase subunit beta

>PSPPH_0070 sensor histidine kinase FimS

>PSPPH_0080 ATP-dependent DNA helicase Rep

>PSPPH_0087 sulfate ABC transporter, permease protein CysW

>PSPPH_0088 sulfate ABC transporter, permease protein CysT

>PSPPH_0089 sulfate-binding protein

>PSPPH_0096 succinate-semialdehyde dehydrogenase I

>PSPPH_0108 lipoprotein, putative

>PSPPH_0152 carbon-nitrogen hydrolase family protein

>PSPPH_0186 citrate transporter

>PSPPH_0192 biopolymer transport protein ExbD

>PSPPH_0196 endoribonuclease L-PSP, putative

>PSPPH_0197 guanosine-3,5-bis(diphosphate) 3-pyrophosphohydrolase

>PSPPH_0198 DNA-directed RNA polymerase subunit omega

>PSPPH_0201 ribonuclease PH

>PSPPH_0203 exodeoxyribonuclease III, putative

>PSPPH_0204 orotate phosphoribosyltransferase

>PSPPH_0207 alginate biosynthesis protein AlgC

>PSPPH_0209 bifunctional phosphopantothenoylcysteine decarboxylase/phosphopantothenate synthase

>PSPPH_0212 50S ribosomal protein L28

>PSPPH_0213 50S ribosomal protein L33

>PSPPH_0240 hypothetical protein

>PSPPH_0245 S1 RNA binding domain protein

>PSPPH_0248 Inhibitor of vertebrate lysozyme precursor

>PSPPH_0249 Protein of unknown function (DUF1328) superfamily

>PSPPH_0256 endonuclease/exonuclease/phosphatase family protein

>PSPPH_0257 thiol:disulfide interchange protein DsbA

>PSPPH_0258 GTPase EngB

>PSPPH_0279 delta-aminolevulinic acid dehydratase

>PSPPH_0280 polyphosphate kinase

>PSPPH_0281 exopolyphosphatase

>PSPPH_0283 amino acid ABC transporter, permease protein

>PSPPH_0284 amino acid ABC transporter, permease protein

>PSPPH_0288 transcription termination factor Rho

>PSPPH_0289 3-octaprenyl-4-hydroxybenzoate carboxy-lyase

>PSPPH_0303 hypothetical protein

>PSPPH_0304 conserved hypothetical protein TIGR02449

>PSPPH_0332 D-methionine ABC transporter, permease protein

>PSPPH_0341 cystine ABC tranporter, permease protein, putative

>PSPPH_0346 hypothetical protein

>PSPPH_0360 glucosyltransferase MdoH

>PSPPH_0365 Sec-independent protein translocase TatC

>PSPPH_0367 twin argininte translocase protein A

>PSPPH_0370 2-polyprenylphenol 6-hydroxylase

>PSPPH_0372 ubiquinone/menaquinone biosynthesis methyltransferase

>PSPPH_0384 arginyl-tRNA synthetase

>PSPPH_0388 NADP-dependent malic enzyme

>PSPPH_0390 type IV pilus biogenesis protein PilM

>PSPPH_0392 type IV pilus biogenesis protein PilO

>PSPPH_0396 3-dehydroquinate synthase

>PSPPH_0427 acyl carrier protein

>PSPPH_0428 acyl carrier protein, putative

>PSPPH_0447 cytochrome b561 family protein

>PSPPH_0448 hypothetical protein

>PSPPH_0454 gluconate transporter family protein

>PSPPH_0455 putative aldolase

>PSPPH_0458 hypothetical protein

>PSPPH_0459 aminotransferase

>PSPPH_0460 dihydroxy-acid dehydratase

>PSPPH_0462 coproporphyrinogen III oxidase

>PSPPH_0463 putative deoxyribonucleotide triphosphate pyrophosphatase

>PSPPH_0464 methionine biosynthesis protein MetW

>PSPPH_0465 homoserine O-acetyltransferase

>PSPPH_0466 YGGT family protein

>PSPPH_0469 twitching motility protein

>PSPPH_0473 aspartate carbamoyltransferase catalytic subunit

>PSPPH_0474 pyrimidine regulatory protein PyrR

>PSPPH_0475 Holliday junction resolvase-like protein

>PSPPH_0476 hypothetical protein

>PSPPH_0480 type IV pilus response regulator PilH

>PSPPH_0493 potassium/proton antiporter

>PSPPH_0544 DNA topoisomerase IV subunit B

>PSPPH_0548 lipoprotein, putative

>PSPPH_0554 flagellar motor protein MotA

>PSPPH_0557 oligoribonuclease

>PSPPH_0561 conserved hypothetical protein TIGR00150

>PSPPH_0564 tRNA delta(2)-isopentenylpyrophosphate transferase

>PSPPH_0565 RNA-binding protein Hfq

>PSPPH_0566 GTP-binding protein HflX

>PSPPH_0568 HflC protein

>PSPPH_0569 ATP phosphoribosyltransferase regulatory subunit

>PSPPH_0574 ribonuclease R

>PSPPH_0576 30S ribosomal protein S6

>PSPPH_0577 30S ribosomal protein S18

>PSPPH_0578 hypothetical protein

>PSPPH_0579 50S ribosomal protein L9

>PSPPH_0594 high affinity branched-chain amino acid ABC transporter, ATP-binding protein

>PSPPH_0619 RNA polymerase sigma factor RpoD

>PSPPH_0620 DNA primase

>PSPPH_0621 30S ribosomal protein S21

>PSPPH_0624 dihydroneopterin aldolase

>PSPPH_0628 hypothetical protein

>PSPPH_0634 4-hydroxythreonine-4-phosphate dehydrogenase

>PSPPH_0660 prophage PSPPH01, putative tail protein

>PSPPH_0680 HIT family protein

>PSPPH_0683 conserved hypothetical protein TIGR00701

>PSPPH_0685 HesB/YadR/YfhF family protein

>PSPPH_0706 ABC-type phosphate/phosphonate transport system periplasmic component

>PSPPH_0712 octylprenyl diphosphate synthase

>PSPPH_0713 50S ribosomal protein L21

>PSPPH_0714 50S ribosomal protein L27

>PSPPH_0715 GTPase ObgE

>PSPPH_0718 30S ribosomal protein S20

>PSPPH_0723 peptidyl-prolyl cis-trans isomerase, FKBP-type

>PSPPH_0739 competence lipoprotein ComL

>PSPPH_0740 Ribosomal large subunit pseudouridine synthase D(Pseudouridine synthase) (Uracil hydrolyase)

>PSPPH_0807 chemotaxis protein CheY

>PSPPH_0809 NADH dehydrogenase

>PSPPH_0811 hypothetical protein

>PSPPH_0816 zinc-binding protein

>PSPPH_0817 dephospho-CoA kinase

>PSPPH_0818 type IV pilus prepilin peptidase PilD

>PSPPH_0831 hypothetical protein

>PSPPH_0835 tellurium resistance protein TerB

>PSPPH_0836 tellurium resistance protein TerC

>PSPPH_0838 tellurium resistance protein TerE

>PSPPH_0854 3-methyl-2-oxobutanoate hydroxymethyltransferase

>PSPPH_0858 sensory box histidine kinase

>PSPPH_0859 hypothetical protein

>PSPPH_0868 penicillin-binding protein 1B

>PSPPH_0872 acetolactate synthase 3 catalytic subunit

>PSPPH_0873 acetolactate synthase 3 regulatory subunit

>PSPPH_0874 ketol-acid reductoisomerase

>PSPPH_0885 hypothetical protein

>PSPPH_0890 carbonic anhydrase

>PSPPH_0989 peptidyl-tRNA hydrolase

>PSPPH_0991 ribose-phosphate pyrophosphokinase

>PSPPH_0994 outer membrane lipoprotein LolB

>PSPPH_0995 TPR domain protein

>PSPPH_0996 glutamyl-tRNA reductase

>PSPPH_1001 hypothetical protein

>PSPPH_1018 uracil phosphoribosyltransferase

>PSPPH_1023 amino acid ABC transporter, periplasmic amino acid-binding protein

>PSPPH_1027 lipoprotein, putative

>PSPPH_1032 hypothetical protein

>PSPPH_1046 endoribonuclease L-PSP family protein

>PSPPH_1058 peptidyl-prolyl cis-trans isomerase, FKBP-type

>PSPPH_1085 PsiE family protein

>PSPPH_1109 alginate biosynthesis protein AlgJ

>PSPPH_1112 alginate biosynthesis protein AlgX

>PSPPH_1113 alginate biosynthesis protein AlgG

>PSPPH_1117 alginate biosynthesis protein Alg8

>PSPPH_1118 alginate biosynthesis protein AlgD

>PSPPH_1121 PhoH-like protein

>PSPPH_1130 amino acid ABC transporter, periplasmic amino acid-binding protein

>PSPPH_1131 amino acid ABC transporter, permease protein

>PSPPH_1132 amino acid ABC transporter, permease protein

>PSPPH_1151 DNA-binding protein HU family

>PSPPH_1153 valyl-tRNA synthetase

>PSPPH_1156 DNA polymerase III subunit chi

>PSPPH_1158 hypothetical protein

>PSPPH_1177 phosphogluconate dehydratase

>PSPPH_1181 glucose ABC transporter, periplasmic glucose-binding protein, putative

>PSPPH_1183 glucose ABC transporter, permease protein, putative

>PSPPH_1185 porin B

>PSPPH_1188 glucose-6-phosphate 1-dehydrogenase

>PSPPH_1195 DNA-binding heavy metal response regulator

>PSPPH_1211 cytochrome o ubiquinol oxidase, subunit I

>PSPPH_1212 cytochrome o ubiquinol oxidase, subunit III

>PSPPH_1213 cytochrome o ubiquinol oxidase, subunit IV

>PSPPH_1214 protoheme IX farnesyltransferase

>PSPPH_1299 S-adenosylmethionine:tRNA ribosyltransferase-isomerase

>PSPPH_1300 queuine tRNA-ribosyltransferase

>PSPPH_1301 preprotein translocase subunit YajC

>PSPPH_1302 preprotein translocase subunit SecD

>PSPPH_1303 preprotein translocase subunit SecF

>PSPPH_1308 iron-sulfur cluster assembly transcription factor IscR

>PSPPH_1309 cysteine desulfurase IscS

>PSPPH_1310 scaffold protein

>PSPPH_1311 iron-sulfur cluster assembly protein IscA

>PSPPH_1314 ferredoxin, 2Fe-2S type, ISC system

>PSPPH_1316 Nucleoside diphosphate kinase (NDK) (NDP kinase)(Nucleoside-2-P kinase)

>PSPPH_1317 radical SAM enzyme, Cfr family

>PSPPH_1320 4-hydroxy-3-methylbut-2-en-1-yl diphosphate synthase

>PSPPH_1321 histidyl-tRNA synthetase

>PSPPH_1322 hypothetical protein

>PSPPH_1323 PQQ enzyme repeat protein

>PSPPH_1324 GTP-binding protein EngA

>PSPPH_1343 hypothetical protein

>PSPPH_1353 signal recognition particle protein

>PSPPH_1354 30S ribosomal protein S16

>PSPPH_1355 16S rRNA-processing protein

>PSPPH_1356 tRNA (guanine-N(1)-)-methyltransferase

>PSPPH_1357 50S ribosomal protein L19

>PSPPH_1374 DNA-binding response regulator, LuxR family

>PSPPH_1386 amino acid ABC transporter, permease protein

>PSPPH_1387 amino acid ABC transporter, permease protein

>PSPPH_1388 amino acid ABC transporter, periplasmic amino acid-binding protein

>PSPPH_1462 ATP-dependent RNA helicase SrmB

>PSPPH_1467 hypothetical protein

>PSPPH_1468 nucleic acid binding protein

>PSPPH_1496 hypothetical protein

>PSPPH_1497 amino acid ABC transporter, permease protein

>PSPPH_1498 Protein of unknown function, DUF485 superfamily

>PSPPH_1499 acetate permease

>PSPPH_1509 asparagine synthase (glutamine-hydrolyzing)

>PSPPH_1513 ABC transporter, ATP-binding protein

>PSPPH_1517 hypothetical protein

>PSPPH_1518 ATP-dependent DNA helicase RecQ

>PSPPH_1519 transcriptional regulator, MarR family

>PSPPH_1537 dihydrodipicolinate synthase

>PSPPH_1538 lipoprotein, putative

>PSPPH_1539 metallo-beta-lactamase family protein

>PSPPH_1545 malic enzyme family protein

>PSPPH_1567 aspartyl-asparaginyl beta-hydroxylase

>PSPPH_1571 recombination associated protein

>PSPPH_1582 peptidyl-prolyl cis-trans isomerase, FKBP-type

>PSPPH_1591 polyamine ABC transporter, permease protein, putative

>PSPPH_1594 ABC transporter, periplasmic substrate-binding protein

>PSPPH_1604 cell division topological specificity factor MinE

>PSPPH_1629 Tetraacyldisaccharide-1-P 4''''-kinase

>PSPPH_1637 Uncharacterized ACR, COG1399

>PSPPH_1638 50S ribosomal protein L32

>PSPPH_1640 malonyl CoA-acyl carrier protein transacylase

>PSPPH_1643 3-oxoacyl-(acyl carrier protein) synthase II

>PSPPH_1648 type IV pilus biogenesis protein PilZ

>PSPPH_1658 acetyl-CoA carboxylase subunit beta

>PSPPH_1661 cvpA family protein

>PSPPH_1662 amidophosphoribosyltransferase

>PSPPH_1678 glutaminyl-tRNA synthetase

>PSPPH_1679 cysteinyl-tRNA synthetase

>PSPPH_1684 ABC transporter, permease protein

>PSPPH_1687 methylenetetrahydrofolate dehydrogenase/methenyltetrahydrofolate cyclohydrolase

>PSPPH_1697 trigger factor

>PSPPH_1698 ATP-dependent Clp protease proteolytic subunit

>PSPPH_1700 ATP-dependent protease La

>PSPPH_1701 DNA-binding protein HU-beta

>PSPPH_1702 peptidyl-prolyl cis-trans isomerase D, putative

>PSPPH_1703 enoyl-(acyl-carrier-protein) reductase

>PSPPH_1728 transcriptional regulator, TetR family

>PSPPH_1766 ureidoglycolate hydrolase

>PSPPH_1767 allantoicase

>PSPPH_1769 polysaccharide deacetylase family protein

>PSPPH_1786 hypothetical protein

>PSPPH_1791 hypothetical protein

>PSPPH_1827 hypothetical protein

>PSPPH_1828 DUF404

>PSPPH_1829 hypothetical protein

>PSPPH_1847 3-ketoacyl-(acyl-carrier-protein) reductase

>PSPPH_1852 glyceraldehyde-3-phosphate dehydrogenase

>PSPPH_1868 anti-sigma factor antagonist

>PSPPH_1915 cation ABC transporter, permease protein

>PSPPH_1917 cation ABC transporter, periplasmic cation-binding protein

>PSPPH_1920 hypothetical protein

>PSPPH_1941 excinuclease ABC subunit B

>PSPPH_1942 glutamyl-tRNA synthetase

>PSPPH_1947 hypothetical protein

>PSPPH_1954 3-isopropylmalate dehydrogenase

>PSPPH_1955 aspartate-semialdehyde dehydrogenase

>PSPPH_1958 galactonate dehydratase

>PSPPH_1961 oxidoreductase, zinc-binding

>PSPPH_1974 type II citrate synthase

>PSPPH_1976 succinate dehydrogenase, cytochrome b556 subunit

>PSPPH_1977 succinate dehydrogenase, hydrophobic membrane anchor protein

>PSPPH_1978 succinate dehydrogenase flavoprotein subunit

>PSPPH_1979 succinate dehydrogenase iron-sulfur subunit

>PSPPH_1980 alpha-ketoglutarate decarboxylase

>PSPPH_1983 succinyl-CoA synthetase subunit beta

>PSPPH_1984 succinyl-CoA synthetase subunit alpha

>PSPPH_1986 lipoprotein, putative

>PSPPH_1987 heat shock protein 90

>PSPPH_1989 3-oxoacyl-(acyl carrier protein) synthase I

>PSPPH_1990 3-hydroxydecanoyl-(acyl carrier protein) dehydratase

>PSPPH_2015 transcriptional regulator, LuxR family

>PSPPH_2016 K+-transporting ATPase, F subunit

>PSPPH_2036 transcription elongation factor GreB

>PSPPH_2038 ABC transporter, ATP-binding protein

>PSPPH_2042 outer membrane lipoprotein OprI

>PSPPH_2061 phosphoenolpyruvate synthase

>PSPPH_2065 crfX protein

>PSPPH_2067 RNA polymerase sigma factor SigX

>PSPPH_2068 OprF

>PSPPH_2082 hypothetical protein

>PSPPH_2109 lipoprotein, putative

>PSPPH_2111 molybdenum cofactor biosynthesis protein B

>PSPPH_2135 threonyl-tRNA synthetase

>PSPPH_2136 translation initiation factor IF-3

>PSPPH_2137 50S ribosomal protein L35

>PSPPH_2138 50S ribosomal protein L20

>PSPPH_2139 phenylalanyl-tRNA synthetase subunit alpha

>PSPPH_2140 phenylalanyl-tRNA synthetase subunit beta

>PSPPH_2141 integration host factor subunit alpha

>PSPPH_2201 hypothetical protein

>PSPPH_2265 alkyl hydroperoxide reductase, C subunit

>PSPPH_2269 transcriptional regulator, putative

>PSPPH_2318 peptidyl-prolyl cis-trans isomerase C

>PSPPH_2328 response regulator, DNA binding component GacA

>PSPPH_2358 papain cysteine protease family protein

>PSPPH_2557 spermidine/putrescine ABC transporter, permease protein, putative

>PSPPH_2618 sulfite reductase (NADPH) hemoprotein, beta-component

>PSPPH_2620 B12-dependent methionine synthase

>PSPPH_2652 ABC transporter, ATP-binding protein

>PSPPH_2677 LexA repressor

>PSPPH_2866 hypothetical protein

>PSPPH_2914 FwdC/FmdC family protein

>PSPPH_2915 glutamine amidotransferase, class-II protein

>PSPPH_2977 transcription antitermination protein, putative

>PSPPH_3015 phosphate ABC transporter, ATP-binding protein

>PSPPH_3016 phosphate ABC transporter, permease protein PtsA

>PSPPH_3017 phosphate ABC transporter, permease protein PstC

>PSPPH_3056 general secretion pathway protein GspM, putative

>PSPPH_3062 general secretion pathway protein GspG

>PSPPH_3077 hypothetical protein

>PSPPH_3089 seryl-tRNA synthetase

>PSPPH_3090 crcB protein, putative

>PSPPH_3096 translation initiation factor IF-1

>PSPPH_3097 ATP-dependent clp protease, ATP-binding subunit ClpA

>PSPPH_3098 ATP-dependent Clp protease adaptor protein ClpS

>PSPPH_3102 tRNA (5-methylaminomethyl-2-thiouridylate)-methyltransferase

>PSPPH_3104 adenylosuccinate lyase

>PSPPH_3105 ycfD protein

>PSPPH_3109 NADH dehydrogenase subunit A

>PSPPH_3110 NADH dehydrogenase subunit B

>PSPPH_3111 bifunctional NADH:ubiquinone oxidoreductase subunit C/D

>PSPPH_3112 NADH dehydrogenase subunit E

>PSPPH_3113 NADH dehydrogenase I subunit F

>PSPPH_3114 NADH dehydrogenase subunit G

>PSPPH_3115 NADH dehydrogenase subunit H

>PSPPH_3116 NADH dehydrogenase subunit I

>PSPPH_3117 NADH dehydrogenase subunit J

>PSPPH_3118 NADH dehydrogenase subunit K

>PSPPH_3119 NADH dehydrogenase subunit L

>PSPPH_3120 NADH dehydrogenase subunit M

>PSPPH_3121 NADH dehydrogenase subunit N

>PSPPH_3173 putative monovalent cation/H+ antiporter subunit C

>PSPPH_3176 putative monovalent cation/H+ antiporter subunit F

>PSPPH_3182 RNA polymerase-binding protein DksA, putative

>PSPPH_3205 hypothetical protein

>PSPPH_3207 DNA topoisomerase I

>PSPPH_3208 hypothetical protein

>PSPPH_3209 3-ketoacyl-CoA thiolase

>PSPPH_3210 multifunctional fatty acid oxidation complex subunit alpha

>PSPPH_3238 hypothetical protein

>PSPPH_3260 carbon storage regulator

>PSPPH_3291 oxidoreductase, molybdopterin-binding

>PSPPH_3294 DNA-binding heavy metal response regulator

>PSPPH_3299 hypothetical protein

>PSPPH_3352 adenine phosphoribosyltransferase

>PSPPH_3353 hypothetical protein

>PSPPH_3354 chemotaxis protein CheW

>PSPPH_3361 chemotaxis protein CheZ

>PSPPH_3362 chemotaxis protein CheY

>PSPPH_3363 flagellar biosynthesis sigma factor

>PSPPH_3364 flagellar synthesis regulator FleN

>PSPPH_3365 flagellar biosynthesis regulator FlhF

>PSPPH_3366 flagellar biosynthesis protein A

>PSPPH_3369 flagellar biosynthesis protein FliQ

>PSPPH_3370 flagellar biosynthesis protein FliP

>PSPPH_3379 flagellar biosynthesis chaperone

>PSPPH_3382 flagellar motor switch protein G

>PSPPH_3383 flagellar MS-ring protein

>PSPPH_3384 flagellar hook-basal body protein FliE

>PSPPH_3387 flagellar regulator FleQ

>PSPPH_3399 flagellar basal body P-ring protein

>PSPPH_3401 flagellar basal body rod protein FlgG

>PSPPH_3402 flagellar basal body rod protein FlgF

>PSPPH_3407 flagellar basal body rod protein FlgC

>PSPPH_3408 flagellar basal body rod protein FlgB

>PSPPH_3412 chemotaxis protein methyltransferase CheR

>PSPPH_3413 chemotaxis protein CheV, putative

>PSPPH_3415 flagellin synthesis, negative regulator, FlgM, putative

>PSPPH_3428 response regulator/TPR domain protein

>PSPPH_3452 hypothetical protein

>PSPPH_3453 DNA-binding response regulator QseB, putative

>PSPPH_3461 L-serine ammonia-lyase

>PSPPH_3504 alginate biosynthesis transcriptional activator

>PSPPH_3510 carbon storage regulator

>PSPPH_3512 alanyl-tRNA synthetase

>PSPPH_3516 hypothetical protein

>PSPPH_3520 arginine N-succinyltransferase, alpha subunit

>PSPPH_3521 bifunctional N-succinyldiaminopimelate-aminotransferase/acetylornithine transaminase protein

>PSPPH_3522 transcriptional regulator ArgR

>PSPPH_3526 arginine/ornithine ABC transporter, permease protein

>PSPPH_3528 acetyl-CoA synthetase

>PSPPH_3538 ribosomal large subunit pseudouridine synthase B

>PSPPH_3539 Protein of unknown function (DUF1289) superfamily

>PSPPH_3541 segregation and condensation protein A

>PSPPH_3543 PHP domain protein

>PSPPH_3544 intracellular septation protein A

>PSPPH_3552 potassium uptake protein TrkH

>PSPPH_3558 bifunctional aconitate hydratase 2/2-methylisocitrate dehydratase

>PSPPH_3563 MoxR-like protein

>PSPPH_3569 exonuclease SbcD

>PSPPH_3593 PspA/IM30 family protein

>PSPPH_3594 hypothetical protein

>PSPPH_3596 hypothetical protein

>PSPPH_3597 Protein of unknown function (DUF1315) superfamily

>PSPPH_3600 inorganic polyphosphate/ATP-NAD kinase

>PSPPH_3631 aminotransferase AlaT

>PSPPH_3634 ATP-dependent RNA helicase, DEAD box family

>PSPPH_3648 elongation factor P

>PSPPH_3662 30S ribosomal protein S1

>PSPPH_3680 hypothetical protein

>PSPPH_3688 hypothetical protein

>PSPPH_3702 cob(I)yrinic acid a,c-diamide adenosyltransferase

>PSPPH_3707 DNA replication initiation factor

>PSPPH_3710 phosphoribosylaminoimidazole synthetase

>PSPPH_3712 hypothetical protein

>PSPPH_3714 hypothetical protein

>PSPPH_3720 hypothetical protein

>PSPPH_3725 DNA-3-methyladenine glycosylase I

>PSPPH_3730 DNA-binding response regulator PhoP

>PSPPH_3766 peptidoglycan-associated lipoprotein

>PSPPH_3767 translocation protein TolB

>PSPPH_3768 tolA protein

>PSPPH_3771 Protein ybgC

>PSPPH_3776 aspartyl-tRNA synthetase

>PSPPH_3779 cold shock domain family protein

>PSPPH_3780 hypothetical protein

>PSPPH_3783 prolyl-tRNA synthetase

>PSPPH_3795 hypothetical protein

>PSPPH_3797 ferredoxin--NADP reductase

>PSPPH_3805 recombinase A

>PSPPH_3814 2-C-methyl-D-erythritol 2,4-cyclodiphosphate synthase

>PSPPH_3821 2-dehydro-3-deoxyphosphooctonate aldolase

>PSPPH_3822 CTP synthetase

>PSPPH_3825 DNA polymerase III subunit alpha

>PSPPH_3828 UDP-N-acetylglucosamine acyltransferase

>PSPPH_3829 (3R)-hydroxymyristoyl-(acyl carrier protein) dehydratase

>PSPPH_3831 outer membrane protein OmpH

>PSPPH_3836 undecaprenyl diphosphate synthase

>PSPPH_3837 ribosome recycling factor

>PSPPH_3838 uridylate kinase

>PSPPH_3839 elongation factor Ts

>PSPPH_3840 30S ribosomal protein S2

>PSPPH_3845 arsC family protein

>PSPPH_3846 tetrahydrodipicolinate succinylase, putative

>PSPPH_3868 ompA family protein

>PSPPH_3873 lysyl-tRNA synthetase

>PSPPH_3874 peptide chain release factor 2, programmed frameshift

>PSPPH_3886 argininosuccinate synthase

>PSPPH_3888 dihydroorotase

>PSPPH_3891 bacterioferritin

>PSPPH_3892 glutaredoxin-related protein

>PSPPH_3899 glycerol kinase

>PSPPH_3903 amino acid ABC transporter, permease protein

>PSPPH_3904 amino acid ABC transporter, permease protein

>PSPPH_3948 ribonuclease III

>PSPPH_3949 hypothetical protein

>PSPPH_3950 signal peptidase I

>PSPPH_3951 GTP-binding protein LepA

>PSPPH_3955 RNA polymerase sigma factor AlgU

>PSPPH_3958 hypothetical protein

>PSPPH_3971 ABC transporter, periplasmic substrate-binding protein

>PSPPH_4013 efflux transporter, RND family, MFP subunit

>PSPPH_4024 formyltetrahydrofolate deformylase

>PSPPH_4025 transcriptional regulator, putative

>PSPPH_4039 pyruvate kinase

>PSPPH_4041 fumarate hydratase, class I, putative

>PSPPH_4056 transcriptional regulator, ArsR family

>PSPPH_4075 DNA-binding response regulator ColR

>PSPPH_4077 chaperonin GroEL

>PSPPH_4078 co-chaperonin GroES

>PSPPH_4103 cell division protein FtsZ

>PSPPH_4105 cell division protein FtsQ, putative

>PSPPH_4108 N-acetylglucosaminyl transferase

>PSPPH_4109 cell division protein FtsW

>PSPPH_4111 phospho-N-acetylmuramoyl-pentapeptide-transferase

>PSPPH_4114 peptidoglycan glycolsyltranferase FtsI

>PSPPH_4115 cell division protein FtsL

>PSPPH_4117 hypothetical protein

>PSPPH_4121 phosphoheptose isomerase

>PSPPH_4122 lipoprotein, putative

>PSPPH_4124 stringent starvation protein A

>PSPPH_4125 30S ribosomal protein S9

>PSPPH_4126 50S ribosomal protein L13

>PSPPH_4133 sulfate adenylyltransferase subunit 2

>PSPPH_4139 UDP-N-acetylglucosamine 1-carboxyvinyltransferase

>PSPPH_4140 toluene tolerance protein Ttg2F, putative

>PSPPH_4144 toluene tolerance proteinTtg2B, putative

>PSPPH_4145 toluene tolerance ABC transporter, ATP-binding protein, putative

>PSPPH_4149 OstA family protein

>PSPPH_4150 ABC transporter, ATP-binding protein

>PSPPH_4151 RNA polymerase factor sigma-54

>PSPPH_4152 ribosomal subunit interface protein

>PSPPH_4163 hypothetical protein

>PSPPH_4173 aspartyl/glutamyl-tRNA amidotransferase subunit C

>PSPPH_4174 aspartyl/glutamyl-tRNA amidotransferase subunit A

>PSPPH_4184 osmotically inducible protein, puative

>PSPPH_4185 polynucleotide phosphorylase/polyadenylase

>PSPPH_4186 30S ribosomal protein S15

>PSPPH_4187 tRNA pseudouridine synthase B

>PSPPH_4188 ribosome-binding factor A

>PSPPH_4189 translation initiation factor IF-2

>PSPPH_4190 transcription elongation factor NusA

>PSPPH_4194 preprotein translocase subunit SecG

>PSPPH_4195 triosephosphate isomerase

>PSPPH_4199 ribosomal RNA large subunit methyltransferase J

>PSPPH_4200 conserved hypothetical protein TIGR00253

>PSPPH_4201 transcription elongation factor GreA

>PSPPH_4202 carbamoyl phosphate synthase large subunit

>PSPPH_4203 carbamoyl phosphate synthase small subunit

>PSPPH_4205 dnaJ protein

>PSPPH_4206 dnaK protein

>PSPPH_4207 heat shock protein GrpE

>PSPPH_4212 Oligoketide cyclase/lipid transport protein

>PSPPH_4213 sodium-dependent transporter, putative

>PSPPH_4251 MFS transporter, phthalate permease family

>PSPPH_4252 5-dehydro-4-deoxyglucarate dehydratase

>PSPPH_4263 dipeptide ABC transporter, permease protein

>PSPPH_4264 dipeptide ABC transporter, permease protein

>PSPPH_4281 hypothetical protein

>PSPPH_4289 hypothetical protein

>PSPPH_4330 carbon starvation protein CstA

>PSPPH_4338 hypothetical protein

>PSPPH_4339 cytochrome d ubiquinol oxidase, subunit II

>PSPPH_4340 cytochrome d ubiquinol oxidase, subunit I

>PSPPH_4344 xanthine/uracil permease family protein

>PSPPH_4348 hypothetical protein

>PSPPH_4357 exonuclease

>PSPPH_4367 endoribonuclease L-PSP family protein

>PSPPH_4374 hypothetical protein

>PSPPH_4385 hypothetical protein

>PSPPH_4388 PhoH family protein

>PSPPH_4389 hypothetical protein

>PSPPH_4397 hypothetical protein

>PSPPH_4399 lipoyl synthase

>PSPPH_4402 D-alanyl-D-alanine carboxypeptidase

>PSPPH_4405 lytic murein transglycosylase B

>PSPPH_4406 rod shape-determining protein RodA

>PSPPH_4408 hypothetical protein

>PSPPH_4409 iojap domain protein

>PSPPH_4411 gamma-glutamyl phosphate reductase

>PSPPH_4421 hypothetical protein

>PSPPH_4424 arginine decarboxylase

>PSPPH_4442 3-dehydroquinate dehydratase

>PSPPH_4443 acetyl-CoA carboxylase biotin carboxyl carrier protein subunit

>PSPPH_4444 acetyl-CoA carboxylase biotin carboxylase subunit

>PSPPH_4445 ribosomal protein L11 methyltransferase

>PSPPH_4447 tRNA-dihydrouridine synthase B

>PSPPH_4448 DNA-binding protein Fis

>PSPPH_4449 bifunctional phosphoribosylaminoimidazolecarboxamide formyltransferase/IMP cyclohydrolase

>PSPPH_4458 precorrin-2 C(20)-methyltransferase

>PSPPH_4459 precorrin-8X methylmutase

>PSPPH_4484 heat shock protein, Hsp70 family

>PSPPH_4489 DNA-binding transcriptional activator

>PSPPH_4501 GTP cyclohydrolase II

>PSPPH_4504 riboflavin synthase subunit beta

>PSPPH_4505 bifunctional 3,4-dihydroxy-2-butanone 4-phosphate synthase/GTP cyclohydrolase II protein

>PSPPH_4506 riboflavin synthase subunit alpha

>PSPPH_4517 lipoprotein, putative

>PSPPH_4520 lipopolysaccharide biosynthesis protein, putative

>PSPPH_4564 bacterioferritin

>PSPPH_4566 50S ribosomal protein L17

>PSPPH_4567 DNA-directed RNA polymerase subunit alpha

>PSPPH_4568 30S ribosomal protein S4

>PSPPH_4569 30S ribosomal protein S11

>PSPPH_4570 30S ribosomal protein S13

>PSPPH_4571 50S ribosomal protein L36

>PSPPH_4572 preprotein translocase subunit SecY

>PSPPH_4573 50S ribosomal protein L15

>PSPPH_4574 50S ribosomal protein L30

>PSPPH_4575 30S ribosomal protein S5

>PSPPH_4576 50S ribosomal protein L18

>PSPPH_4577 50S ribosomal protein L6

>PSPPH_4578 30S ribosomal protein S8

>PSPPH_4579 30S ribosomal protein S14

>PSPPH_4580 50S ribosomal protein L5

>PSPPH_4581 50S ribosomal protein L24

>PSPPH_4583 30S ribosomal protein S17

>PSPPH_4584 50S ribosomal protein L29

>PSPPH_4585 50S ribosomal protein L16

>PSPPH_4586 30S ribosomal protein S3

>PSPPH_4587 50S ribosomal protein L22

>PSPPH_4588 30S ribosomal protein S19

>PSPPH_4589 50S ribosomal protein L2

>PSPPH_4590 50S ribosomal protein L23

>PSPPH_4591 50S ribosomal protein L4

>PSPPH_4592 50S ribosomal protein L3

>PSPPH_4595 elongation factor G

>PSPPH_4596 30S ribosomal protein S7

>PSPPH_4597 30S ribosomal protein S12

>PSPPH_4598 DNA-directed RNA polymerase subunit beta'

>PSPPH_4599 DNA-directed RNA polymerase subunit beta

>PSPPH_4600 50S ribosomal protein L7/L12

>PSPPH_4602 50S ribosomal protein L1

>PSPPH_4603 50S ribosomal protein L11

>PSPPH_4605 preprotein translocase subunit SecE

>PSPPH_4622 transcriptional regulator, TetR family

>PSPPH_4653 hypothetical protein

>PSPPH_4666 aldehyde dehydrogenase family protein

>PSPPH_4667 ethanolamine ammonia-lyase, heavy subunit

>PSPPH_4672 inorganic pyrophosphatase

>PSPPH_4704 coenzyme PQQ synthesis protein PqqA

>PSPPH_4705 pyrroloquinoline quinone biosynthesis protein PqqB

>PSPPH_4706 pyrroloquinoline quinone biosynthesis protein PqqC

>PSPPH_4710 hypothetical protein

>PSPPH_4721 biotin synthase

>PSPPH_4731 predicted kinase

>PSPPH_4742 hypothetical protein

>PSPPH_4748 glycine/betaine/L-proline ABC transporter, permease protein

>PSPPH_4751 sarcosine oxidase, beta subunit

>PSPPH_4752 sarcosine oxidase, delta subunit

>PSPPH_4754 sarcosine oxidase, gamma subunit

>PSPPH_4760 hypothetical protein

>PSPPH_4766 choline dehydrogenase

>PSPPH_4767 betaine aldehyde dehydrogenase

>PSPPH_4768 transcriptional regulator BetI

>PSPPH_4774 thiazole synthase

>PSPPH_4775 sulfur carrier protein ThiS

>PSPPH_4778 RNA polymerase factor sigma-32

>PSPPH_4780 cell division ATP-binding protein FtsE

>PSPPH_4782 peptidase, M16 family

>PSPPH_4795 hypothetical protein

>PSPPH_4805 oxidoreductase FAD-binding domain/oxidoreductase NAD-binding domain/2Fe-2S iron-sulfur cluster binding domain protein

>PSPPH_4806 Rieske 2Fe-2S domain protein

>PSPPH_4813 renal dipeptidase family protein

>PSPPH_4816 fructose-1,6-bisphosphate aldolase

>PSPPH_4820 D-erythrose 4-phosphate dehydrogenase

>PSPPH_4821 transketolase

>PSPPH_4823 S-adenosylmethionine synthetase

>PSPPH_4846 thiamine biosynthesis protein ThiI

>PSPPH_4847 glutamine synthetase, type I

>PSPPH_4852 nitrogen regulation protein NR(II)

>PSPPH_4861 histidine transporter, periplasmic histidine-binding protein

>PSPPH_4862 histidine ABC transporter, permease protein, putative

>PSPPH_4863 histidine ABC transporter, ATP-binding protein, putative

>PSPPH_4865 histidine ammonia-lyase

>PSPPH_4867 imidazolonepropionase

>PSPPH_4870 Cd(II)/Pb(II)-responsive transcriptional regulator

>PSPPH_4875 dinucleoside polyphosphate hydrolase

>PSPPH_4880 ribose-5-phosphate isomerase A

>PSPPH_4885 D-3-phosphoglycerate dehydrogenase

>PSPPH_4893 putrescine ABC transporter, ATP-binding protein

>PSPPH_4897 glutamine synthetase

>PSPPH_4898 glutamine synthetase

>PSPPH_4904 aliphatic sulfonates ABC transporter, permease protein

>PSPPH_4914 glutaredoxin 3

>PSPPH_4923 1-(5-phosphoribosyl)-5-[(5-phosphoribosylamino)methylideneamino] imidazole-4-carboxamide isomerase

>PSPPH_4925 imidazole glycerol phosphate synthase subunit HisH

>PSPPH_4926 imidazoleglycerol-phosphate dehydratase

>PSPPH_4928 MFS permease-like protein

>PSPPH_4930 A/G-specific adenine glycosylase

>PSPPH_4931 hypothetical protein

>PSPPH_5000 nitrogen regulatory protein P-II

>PSPPH_5008 diaminopimelate decarboxylase

>PSPPH_5009 lipoprotein LppL-related protein

>PSPPH_5012 nucleoside diphosphate kinase regulator

>PSPPH_5017 ribosomal protein S6 modification protein

>PSPPH_5018 possible ribosomal protein S6 modification protein

>PSPPH_5022 phosphoenolpyruvate carboxykinase

>PSPPH_5042 transcriptional regulator, AsnC family

>PSPPH_5058 HAD-superfamily hydrolase, subfamily IA

>PSPPH_5084 hypothetical protein

>PSPPH_5102 hypothetical protein

>PSPPH_5109 DNA-binding protein HU family

>PSPPH_5114 phosphate regulon transcriptional regulatory protein PhoB

>PSPPH_5119 phosphate transport system regulatory protein PhoU

>PSPPH_5125 cytosolic long-chain acyl-CoA thioester hydrolase family protein

>PSPPH_5128 hypothetical protein

>PSPPH_5130 phosphoribosylaminoimidazole carboxylase, catalytic subunit

>PSPPH_5145 transcriptional regulator, RpiR family

>PSPPH_5147 DNA-dependent helicase II

>PSPPH_5148 Tim44-like domain family

>PSPPH_5149 hypothetical protein

>PSPPH_5153 hypothetical protein

>PSPPH_5177 hypothetical protein

>PSPPH_5190 sugar ABC transporter, permease protein

>PSPPH_5203 D-fructose-6-phosphate amidotransferase

>PSPPH_5206 F0F1 ATP synthase subunit epsilon

>PSPPH_5207 F0F1 ATP synthase subunit beta

>PSPPH_5208 F0F1 ATP synthase subunit gamma

>PSPPH_5209 F0F1 ATP synthase subunit alpha

>PSPPH_5210 F0F1 ATP synthase subunit delta

>PSPPH_5211 F0F1 ATP synthase subunit B

>PSPPH_5212 F0F1 ATP synthase subunit C

>PSPPH_5213 F0F1 ATP synthase subunit A

>PSPPH_5214 F0F1 ATP synthase subunit I

>PSPPH_5216 chromosome partitioning protein ParA

>PSPPH_5217 glucose-inhibited division protein B

>PSPPH_5221 conserved hypothetical protein TIGR00278

>PSPPH_5222 ribonuclease P

>PSPPH_5223 50S ribosomal protein L34
