# Supplementary material for: Comparative Genome Analysis Provides Insights into the Evolution and Adaptation of Pseudomonas syringae pv. aesculi on Aesculus hippocastanum
Source: PLoS One. 2010 Apr 19;5(4):e10224. doi: 10.1371/journal.pone.0010224 (PMC2856684; doi:10.1371/journal.pone.0010224)
Supplement: Text S1 — Text for supporting information. (0.04 MB DOC) [file pone.0010224.s008.doc]

# Supporting Information

## Two type VI secretion systems present in I-Pae but absent in E-Pae

The Type VI secretion system (T6SS) is a recently recognized, novel mechanism thought to be involved in protein secretion across host cell membranes [1]. The genome assembly of I-*Pae* encoded components of two distinct T6SS [1]: one showed significant amino acid sequence similarity to a T6SS in *Pph* 1448A. The other was similar to that of a T6SS in *Pto* DC3000 (Table S2) and was also conserved in *Pta* 11528 [2]. In contrast, E-*Pae* lacked homologues of several key components of both T6SS and so apparently does not encode a functional T6SS. The homologues of VasA, VasB and VasK present in E-*Pae* (Table S3) represent molecular relics of the degenerate T6SS and are probably no longer functional. Both I-*Pae* and E-*Pae* encode Hcp proteins (recognised by their match to the DUF796, Pfam:PF05638) and VgrG proteins (matching DUF586, Pfam:PF05424) (Table S2).

The T6SS has been most studied in the symbiont *Rhizobium leguminosarum* and the pathogens *Vibrio cholerae*, *Salmonella enterica* and *Pseudomonas aeruginosa* where it is implicated in interactions with a eukaryotic host. Lindeberg *et al.* [3] identified two T6SS-encoding gene clusters in *Pto* DC3000 and concluded that, since these were absent from *Psy* B728a and *Pph* 1448A, the function of T6SS in *P. syringae* must be strain-specific. However, on the basis of sequence similarity, Arnold *et al*. [4] identified clusters in *Psy* B728a and *Pph* 1448A with the potential to encode T6SS, with little sequence similarity between the two. The biological significance of T6SS in *P. syringae* is not known, but based on the precedent from other bacteria, it might be involved in interactions with eukaryotic cells. Potentially, the *P. syringae* T6SS may help the pathogen to infect plants [5, 6] or to be used as an anti-predation mechanism, targeting bacteriovorous organisms such as nematodes and amoeba [7]; further study of this gene system is needed to determine the ecological role and discern whether it could be one factor that has promoted rapid spread in the environment or altered pathogenesis.

## I-Pae encodes a microcin that is absent from E-Pae

Microcins are small polypeptide antibiotics produced by diverse enteric bacteria [8-12]. One of these, MccB17, produced by *E. coli*, exerts antibacterial activity by inhibiting DNA gyrase and consequently DNA elongation. Functionally, the MccB17 production operon can be divided into two parts, *mcbABCD* required for production and *mcbEFG* required for resistance. The *mcb*A encodes the inactive precursor that is processed by the MccB17 synthetases McbB, McbC and McbD. McbE and McbF serve two functions: they are involved in MccB17 secretion, but also contribute to self-immunity towards MccB17 acting together with McbG. A 5 kb region restricted to I-*Pae* had sequence similarity to a microcin B17 (MccB17) -encoding operon produced by *E. coli* and encoding homologues of McbBCDEFG (genes 1410-1404) (Figure S2). In *E. coli*, the MccB17 precursor MccA is an heptapeptide with sequence MRTGNAN. Immediately upstream of *mcbBCDEFG* in I-*Pae* was an open reading frame encoding the octapeptide MCKNLFKN (gene 1411), which probably serves as the microcin precursor in I-*Pae*. Microcin synthesis also requires the enterobactin gene *entF*, a homologue of which was found in I-*Pae* (gene 3669). In I-*Pae* immediately downstream of the genes for microcin processing were homologues of *mcbF* and *mcbG* (genes 1405 and 1404, respectively), which, in *E. coli*, encode proteins required for immunity to microcin B17. These two immunity genes were conserved in E-*Pae* (genes 4701 and 4702) despite the absence of microcin-processing enzymes.

## I-Pae encodes a novel methionine sulphoxide absent from E-Pae

Peptide methionine sulphoxide reductase (MSR) is an enzyme that reverses the oxidation of methionine in polypeptides that occurs under mild oxidizing conditions [13]. MSR has been implicated in resistance to peroxide, nitrite, and other oxidative species in post-translational regulation of protein function and may have a role in bacterial adherence and motility [13]. Orthologues of two methionine sulphoxide reductase (MSR) enzymes encoded by *Pph* 1448A (PSPPH_4802 [MsrA] and PSPPH_3630 [MsrB]) were encoded in the genome assemblies of both I-*Pae* and E-*Pae*. However, I-*Pae* encoded an additional MSR (gene 2670) that was absent from E-*Pae* and was phylogenetically distinct from other *P. syringae* MsrA and MsrB. I-*Pae* gene 2670 most closely resembled predicted MsrA (PFLU_4277) from *P. fluorescens* SBW25.

## Quality control of de novo draft genome assemblies

To assess the quality of the *de novo* assemblies, we checked for the presence of intact copies of highly conserved *P. syringae* genes. We assembled a collection of 683 *Pph* 1448A genes each of which has a single homologue in both *Pto* DC3000 and *Psy* B728a. Each of these *Pph* 1448A genes shares at least 90% nucleotide sequence identity over its full length in both *Pto* and *Psy*. Using BLASTN, we checked for a homologue, with at least 90% sequence identity and coverage of at least 99% of its length, of each of these genes in our *de novo* assemblies. In the E-*Pae* genome assembly, we recovered full-length matches to 676 of the 683 genes (*i.e.* 98.97%). In the I-*Pae* assembly, we recovered 636 of the 683 genes (*i.e.* 97.21%). The seven genes that were not recovered in E-*Pae* were: PSPPH_1181 (glucose ABC transporter periplasmic glucose-binding protein), PSPPH_5203 (D-fructose-6-phosphate amidotransferase), PSPPH_1961 (oxidoreductase, zinc-binding), PSPPH_3207 (DNA topoisomerase I), PSPPH_5109 (DNA-binding protein HU family), PSPPH_1303 (preprotein translocase subunit SecF), PSPPH_4564 (bacterioferritin). In the case of three of these genes (PSPPH_5203, PSPPH_1961 and PSPPH_3207) the *de novo* assembly had failed to resolve repetitive sequences within the genes, resulting in splitting of each gene over a pair of adjacent contigs (within a scaffold). In the case of PSPPH_1303, PSPPH_4564 and PSPPH_5109, the genes were actually intact in the assembly. Their failure to be recovered was an artefact of the blastn searches; because the *Pae* genes diverged from the *Pph* 1448A genes by just one or two nucleotides in a 3’ codon, blastn reported a truncated hit. PSPPH_1181, is conserved in *Pto* and *Psy*, but is absent from the E-*Pae* assembly. Independently of the assembly, we found that only 74% of this gene was covered in the MAQ alignments of E-*Pae* Illumina reads against the *Pph* 1448A genome, whereas most (679 of 683) of the conserved genes are covered over at least 85% of their length. This suggests that PSPPH_1181 might be at least partially absent or highly divergent in *Pae*. Overall, these results strongly suggest that in our *de novo* assemblies nearly all the gene-space is intact, albeit that a few genes are split over two contigs.

## References for Supporting Information

1. Filloux A, Hachani A, Bleves S (2008) The bacterial type VI secretion machine: yet another player for protein transport across membranes. Microbiology 154: 1570-83.
2. Studholme DJ, Gimenez Ibanez S, Maclean D, Dangl JL, Chang JH, *et al.* (2009) A draft genome sequence and functional screen reveals the repertoire of type III secreted proteins of *Pseudomonas syringae* pathovar *tabaci* 11528. BMC Genomics 10:395.
3. Lindeberg M, Myers CR, Collmer A, Schneider DJ (2008) Roadmap to new virulence determinants in *Pseudomonas syringae*: insights from comparative genomics and genome organization. Mol Plant Microbe Interact 21: 685-700.
4. Arnold DL, Godfrey SAC, Jackson RW (2009) *Pseudomonas syringae* Genomics Provides Important Insights to Secretion Systems, Effector Genes and the Evolution of Virulence. In Plant Pathogenic Bacteria: Genomics and Molecular Biology. Ed. Jackson, RW. Caister Academic Press. ISBN: 978-1-904455-37-0. Chapter 10.
5. Bladergroen MR, Badelt K, Spaink HP (2003) Infection-blocking genes of a symbiotic *Rhizobium leguminosarum* strain that are involved in temperature-dependent protein secretion. Mol Plant Microbe Interact 16: 53-64.
6. Liu H, Coulthurst SJ, Pritchard L, Hedley PE, Ravensdale M, *et al*. (2008)Quorum sensing coordinates brute force and stealth modes of infection in the plant pathogen *Pectobacterium atrosepticum*. PLoS Pathog 4:e1000093
7. Pukatzki S, Ma AT, Sturtevant D, Krastins B, Sarracino D, *et al.* (2006) Identification of a conserved bacterial protein secretion system in *Vibrio cholerae* using the *Dictyostelium* host model system. Proc Natl Acad Sci USA 103: 1528-1533.
8. Duquesne S, Destoumieux-Garzón D, Peduzzi J, Rebuffat S (2007) Microcins, gene-encoded antibacterial peptides from enterobacteria Nat Prod Rep 24: 708-734.
9. Genilloud O, Moreno F, Kolter R (1989) DNA sequence, products, and transcriptional pattern of the genes involved in production of the DNA replication inhibitor microcin B17. J Bacteriol 171: 1126-35.
10. Jack RW, Jung G (2000) Lantibiotics and microcins: polypeptides with unusual chemical diversity. Curr Opin Chem Biol. 4: 310-317.
11. Pons AM, Lanneluc I, Cottenceau G, Sable S (2002) New developments in non-post translationally modified microcins. Biochimie 84: 531-537.
12. Severinov K, Semenova E, Kazakov A, Kazakov T, Gelfand MS (2007) Low-molecular-weight post-translationally modified microcins. Mol Microbiol 65: 1380-1394.
13. Brot N, Weissbach H (2000) Peptide methionine sulfoxide reductase: biochemistry and physiological role. Biopolymers 55: 288-296.
14. Weissbach H, Resnick L, Brot N (2005) Methionine sulfoxide reductases: history and cellular role in protecting against oxidative damage. Biochim Biophys Acta 1703: 203-212.
15. Mougous JD, Cuff ME, Raunser S, Shen A, Zhou M, *et al*. (2006) A virulence locus of *Pseudomonas aeruginosa* encodes a protein secretion apparatus. Science 312: 1526-1530.
16. Altschul SF, Gish W, Miller W, Myers EW, Lipman DJ (1990) Basic local alignment search tool. J Mol Biol 215: 403-410.
17. Katoh K, Misawa K, Kuma K, Miyata T (2002) MAFFT: a novel method for rapid multiple sequence alignment based on fast Fourier transform. Nucleic Acids Res 30: 3059-3066.
18. Howe K, Bateman A, Durbin R (2002) QuickTree: building huge Neighbour-Joining trees of protein sequences. Bioinformatics 18: 1546-1547.
